# Supplementary material for: Poxviruses capture host genes by LINE-1 retrotransposition
Source: eLife. 2022 Sep 7;11:e63332. doi: 10.7554/eLife.63332 (PMC9578705; doi:10.7554/eLife.63332)
Supplement: Supplementary file 1. — Accession numbers for sequences used in phylogenetic analysis shown in Figure 1B. Amino acid alignment used in phylogenetic analysis shown in Figure 1B. Supplementary sequences. [file elife-63332-supp1.docx]

**Supplementary Materials - Poxviruses capture host genes by LINE-1 retrotransposition**

Table S1. Accession numbers for sequences used in phylogenetic analysis shown in Figure 1B

| **Organism** | **protein** | **Accession number** |
| --- | --- | --- |
| Cowpox strain Ger14Cat1 | vGAAP/TMBIM4 | SNB58186.1 |
| Cowpox strain Ger10Rac | vGAAP/TMBIM4 | SNB48400.1 |
| Cowpox strain Ger02MKY | vGAAP/TMBIM4 | ADZ30397.1 |
| Cowpox strain Ratpox09 | vGAAP/TMBIM4 | CRL86727.1 |
| Cowpox strain Ger17vFME | vGAAP/TMBIM4 | SPN68348.1 |
| Vaccinia strain EvansCDS | vGAAP/TMBIM4 | AAV98625.1 |
| Cowpox strain Finland_2000_MAN | vGAAP/TMBIM4 | ADZ29327.1 |
| Cowpox strain Kost15Hs | vGAAP/TMBIM4 | AQQ13071.1 |
| Vaccinia strain Lister | vGAAP/TMBIM4 | Q49P94.1 |
| Cowpox strain Aus99cat | vGAAP/TMBIM4 | ADZ24213.1 |
| Vaccinia strain USSR_CDS | vGAAP/TMBIM4 | AAW21699.1 |
| Camelpox strain CMS | vGAAP/TMBIM4 | AAG37461.1 |
| Camelpox strain Negev16 | vGAAP/TMBIM4 | QCW07309.1 |
| Camelpox strain M96_Kazakstan | vGAAP/TMBIM4 | NP_570396.1 |
| Cowpox strain GRI-90 | vGAAP/TMBIM4 | CAD90752.1 |
| Cowpox strain FM2292 | vGAAP/TMBIM4 | CRL87022.1 |
| Cowpox strain Ger07Vole | vGAAP/TMBIM4 | SNB49536.1 |
| Cowpox strain Ger91-3 | vGAAP/TMBIM4 | ABD97561.1 |
| Monkeypox strain USA03_044 | vGAAP/TMBIM4 | AAY96807.1 |
| Monkeypox strain Cote d'Ivoire 1971 | vGAAP/TMBIM4 | AKG51345.1 |
| Monkeypox strain DRC_07 | vGAAP/TMBIM4 | AGR36454.1 |
| Monkeypox strain Zaire96 | vGAAP/TMBIM4 | NP_536611.1 |
| Monkeypox strain Congo03 | vGAAP/TMBIM4 | AAY97191.1 |
| Scale Drop Disease Iridovirus | TMBIM4 | YP_009163856.1 |
| Harpseal herpes virus | TMBIM4 | AJG42933.1 |
| Homo sapiens | TMBIM4 | NP_057140.2 |
| Mus musculus | TMBIM4 | NP_080893.1 |
| Rattus Norvigicus | TMBIM4 | NP_954547.1 |
| Bos taurus | TMBIM4 | NP_001014914.1 |
| Ovis aries | TMBIM4 | XP_014950252.2 |
| Grizzly bear | TMBIM4 | XP_026355879.1 |
| CA Sea lion | TMBIM4 | XP_027451274.1 |
| Stellar sea lion | TMBIM4 | XP_027966737.1 |
| Weddell seal | TMBIM4 | XP_006731256.1 |
| Northern fur seal | TMBIM4 | XP_025738732.1 |
| Ghost shark | TMBIM4 | NP_001279731.1 |
| Bettafish | TMBIM4 | XP_029017613.1 |
| Yellow perch | TMBIM4 | XP_028426613.1 |
| Gilt head Bream | TMBIM4 | XP_030296356.1 |
| Homo sapiens | TMBIM3 | NP_000828.1 |
| Mus musculus | TMBIM3 | NP_075657.1 |
| Rattus Norvigicus | TMBIM3 | NP_695220.4 |
| Bos taurus | TMBIM3 | NP_001032682.1 |
| Ovis aries | TMBIM3 | NP_001265493.1 |
| Grizzly bear | TMBIM3 | XP_026344576.1 |
| CA Sea lion | TMBIM3 | XP_027465497.1 |
| Weddell seal | TMBIM3 | XP_006745469.1 |
| Northern fur seal | TMBIM3 | XP025727080.1 |
| Whale shark | TMBIM3 | XP020385145.1 |
| Bettafish | TMBIM3 | XP_029023514.1 |
| Yellow perch | TMBIM3 | XP_028453020.1 |
| Gilt head Bream | TMBIM3 | XP_030268118.8 |
| Homo sapiens | TMBIM2 | NP_036438.2 |
| Mus musculus | TMBIM2 | NP_082500.2 |
| Rattus Norvigicus | TMBIM2 | NP_653357.1 |
| Bos taurus | TMBIM2 | NP_001068886.1 |
| Ovis aries | TMBIM2 | XP_0040066421.1 |
| Grizzly bear | TMBIM2 | XP_026357704.1 |
| CA Sea lion | TMBIM2 | XP_027450780.1 |
| Weddell seal | TMBIM2 | XP_006729299.1 |
| Northern fur seal | TMBIM2 | XP_025739188.1 |
| Whale shark | TMBIM2 | XP_020373190.1 |
| Bettafish | TMBIM2 | XP_029006890.1 |
| Yellow perch | TMBIM2 | XP_028437735.1 |
| Atlantic sea bass | TMBIM2 | XP_018521446.1 |
| Gilt head bream | TMBIM2 | XP_030278166.1 |
| Danio rerio | TMBIM2 | XP_005163554.1 |
| Homo sapiens | TMBIM1 | NP_001308356.1 |
| Mus musculus | TMBIM1 | NP_081430.3 |
| Rattus Norvigicus | TMBIM1 | NP_001007714.1 |
| Bos taurus | TMBIM1 | NP_991367.1 |
| Ovis aries | TMBIM1 | XP_004004971.1 |
| CA Sea lion | TMBIM1 | XP_027443873.1 |
| Weddell seal | TMBIM1 | XP_006750605.1 |
| Northern fur seal | TMBIM1 | XP_025747486.1 |
| Ghost shark | TMBIM1 | XP_007882730.1 |
| Bettafish | TMBIM1 | XP_028986890.1 |
| Yellow perch | TMBIM1 | XP_028448525.1 |
| Atlantic sea bass | TMBIM1 | XP_018532965 |
| Gilt head bream | TMBIM1 | XP_030257449.1 |
| Danio rerio | TMBIM1 | NP_001005992.2 |
| Homo sapiens | TMBIM5 | NP_055209.2 |
| Mus musculus | TMBIM5 | NP_001186051.1 |
| Rattus Norvigicus | TMBIM5 | NP_001005908.1 |
| Bos taurus | TMBIM5 | NP_001029224.1 |
| Ovis aries | TMBIM5 | XP_004021578.1 |
| Grizzly bear | TMBIM5 | XP_026360365.1 |
| CA Sea lion | TMBIM5 | XP_027452399.1 |
| Monk seal | TMBIM5 | XP_021555821.1 |
| Northern fur seal | TMBIM5 | XP_025736444.1 |
| Ghost shark | TMBIM5 | AFK11640.1 |
| Bettafish | TMBIM5 | XP_028990283.1 |
| Yellow perch | TMBIM5 | XP_028423643.1 |
| Atlantic sea bass | TMBIM5 | XP_18554801.1 |
| Danio rerio | TMBIM5 | NP_956885.1 |
| Homo sapiens | TMBIM6 | NP_003208.2 |
| Mus musculus | TMBIM6 | NP_001164505.1 |
| Rattus Norvigicus | TMBIM6 | NP_062254.2 |
| Bos taurus | TMBIM6 | NP_001069882.1 |
| Ovis aries | TMBIM6 | XP_004006423.2 |
| Grizzly bear | TMBIM6 | XP_026357713.1 |
| CA Sea lion | TMBIM6 | XP_027449007.1 |
| Weddell seal | TMBIM6 | XP_006729302.1 |
| Northern fur seal | TMBIM6 | XP_025738444.1 |
| Ghost shark | TMBIM6 | XP_007910207.1 |
| Bettafish | TMBIM6 | XP_029013526.1 |
| Yellow perch | TMBIM6 | XP_028439299.1 |
| Atlantic sea bass | TMBIM6 | XP_018531358.1 |
| Gilt head bream | TMBIM6 | XP_030277962.1 |

Phylip alignment for sequences used in phylogenetic analysis shown in Figure 1B

109 436

VACLister ---------------------------------------------------

CML_Ngv16 ---------------------------------------------------

CMLV_Orig ---------------------------------------------------

CMLV_CMS_ ---------------------------------------------------

CPX_Ger07 ---------------------------------------------------

CPXFM2292 ---------------------------------------------------

CPX_Gri90 ---------------------------------------------------

CPX_Aus99 ---------------------------------------------------

CPX_Kos15 ---------------------------------------------------

CPX_Fin00 ---------------------------------------------------

CPX_Ger17 ---------------------------------------------------

CPX_Rat09 ---------------------------------------------------

CPX_Ger91 ---------------------------------------------------

CPX_Ger02 ---------------------------------------------------

CPX_Ger14 ---------------------------------------------------

CPX_Ger10 ---------------------------------------------------

CPX_Ger80 ---------------------------------------------------

CPX_Ger98 ---------------------------------------------------

MPX_USA03 ---------------------------------------------------

MPX_Ivr71 ---------------------------------------------------

MPX_Zre96 ---------------------------------------------------

MPX_Con03 ---------------------------------------------------

MPX_DRC07 ---------------------------------------------------

SDDIridoV ---------------------------------------------------

HSherpesV ---------------------------------------------------

Human_LG4 ---------------------------------------------------

Mouse_LG4 ---------------------------------------------------

NRat_LFG4 ---------------------------------------------------

Cow_LFG4_ ---------------------------------------------------

Sheep_LG4 ---------------------------------------------------

GrizzlyL4 ---------------------------------------------------

CAclionL4 ---------------------------------------------------

StClionL4 ---------------------------------------------------

WeddellL4 ---------------------------------------------------

Nfurseal4 ---------------------------------------------------

GShark_L4 ---------------------------------------------------

betta_LG4 ---------------------------------------------------

YlwPrchL4 ---------------------------------------------------

GHbreamL4 ---------------------------------------------------

Human_LG1 MSHEKS-FLVSGDNYPPPNPGYPGGPQPP--MPPYAQPPYPGAPYPQPPFQ

Mouse_LG1 MSHEKS-FLVSGDSYPPQNI---VGPQAP--MPPYVQAPYPGAPYPQAPFQ

NRat_LFG1 MSHEKS-FLVSGDSYPPPNPGYPVGPQAP--MPPYVQPPYPGAPYPQAAFQ

Cow_LFG1_ MSHEKS-FLVSGDSYPPPNPGYPGGPQPS--MAPY-----PGAPYPQAPFQ

Nfseal_L1 MPHEKS-FLVSGDSYPPPNSGYPGGPQPS--MPPY-----PVAPYPQAPFQ

WeddellL1 MPHEKS-FLVSGDSYPPPNPGYPGGPQPS--MPPY-----PGAPYPQAPFQ

CAslionL1 MPHEKS-FLVSGDSYPPPNSGYPGGPQPS--MPPY-----PVAPYPQAPFQ

Sheep_LG1 MSHEKS-FLVSGDSYPPPNPGYPEGPQPS--MAPY-----PGAPYPQAAFQ

GrizzlyL1 MPHEKS-FLVSGDSYPPPNPGYPGGPQPS--MPPY-----PGAPYPQAPFQ

WhSharkL1 MSQEKT-YLVSGD---PQQPPY---PRNEMSYGNDHYPQQP-PPYGQPVFP

Betta_LG1 MSQDKSGYPVMGETNPLHSNMY-GPPQPGFGMPPPNYSQAPGGPYPPA---

GHbreamL1 MSQDKSGYPIMGETNPLHNNVY-GPPQPGFGMPPPNYSQAPGGPYPPA---

YlwPrchL1 MSQEKSGYPVMGENNPLHNNVY-GPPQPGFGMPPPNYSQAPGGPYPPA---

Human_LG2 MTQGKLSVANKAPGTEGQQ-QVH---GEKK--------EAPAVPSAPPSYE

Mouse_LG2 MTQGKLSVANKAPGTEGQQHQAN---GEKK--------DAPAVPSAPPSYE

NRat_LFG2 MTQGKLSVANKAPGTEGQQ-QAN---GEKK--------DAPAVPSAPPSYE

Cow_LFG2_ MTQGKLSVANKAPGTEGQQ-QAN---GEKK--------ETPAVPSAPPSYE

Sheep_LG2 MTQGKLSVANKAPGTEGQQ-QAN---GEKK--------ETPAVPSAPPSYE

GrizzlyL2 MTQGKISVANKAPGTEGQQ-QAN---GEKK--------ETPAVPSAPPSYE

WeddellL2 MTQGKISVANKAPGTEGQQ-QAN---GEKK--------ETPAVPSAPPSYE

Nfseal_L2 MTQGKISVANKAPGTEGQQ-QAN---GEKK--------ETPAVPSAPPSYE

CAslionL2 MTQGKISVANKAPGTEGQQ-QAN---G------------------------

WhSharkL2 ---------------------------------------------------

betta_LG2 MKKGKVSDANE-----------------------------------PPSYQ

Asbass_L2 MTQGKLSLANKA--TNGSS---S---GQAL--------VAPA----PPSYE

Ylwprh_L2 MTKGKLSLANKA--TNGSF---S---EEAL--------VSPA----PPSYQ

GHbreamL2 MTQGKLSLANKS---NDSS---S---GQAL--------GTPS----PPSYE

Zfish_LG2 M----------------------------------------SLPAAPPSYA

Human_LG3 MSNPSAPPPYED-RNPL-------YPGP---PPPGGYGQPSVLPGGYPAYP

Mouse_LG3 MSNPSAPPPYED-HNPL-------YPGS---PPPGGYGQPSVLPGGYPAYP

NRat_LFG3 MSNPTAPPPYED-HNPL-------YPGR---PPPGGYGQPSVLPGGYPAYP

Cow_LFG3_ MSHPSAPPPYED-RSPL-------YPGS---PPPGGYGQPSVMPGGYP---

Sheep_LG3 MSHASAPPPYED-RSPL-------YPGS---LPPGGYGQPSVVPGGYP---

Nfseal_L3 MSNPSAPPPYED-RNPL-------YPGS---PPQGGYAQPSVLPGGYPPYP

CAslionL3 MSNPSAPPPYED-RNPL-------YPGS---PPQGGYAQPSVLPGGYPPYP

WeddellL3 MSNPSAPPPYED-RNPL-------YPGS---PPQGGYAQPFVLPGGYP---

GhSharkL3 MSRPTAPPSYEEATDLVMLPVHGEYPGNTVYPLPGSY-----SPTGKPGYP

Betta_LG3 MSRSDYPPGYEDCHGPL-------YP------PQGGNYPAP-PPYGFPGY-

Asbass_L3 MSRSDYPPGYDDSHGPL-------YA------PQGGSYPPP-PAYGFPAY-

GHbreamL3 MSRSDYPPGYDDSRGPM-------YA------PQGGNYPPP-PAYGFPAY-

YlwPrchL3 MSRSDYPPGYDDSRDLL-------YA------PQGGNYPPP-PAYGFPGF-

Zfish_LG3 MSRSDFPPSYDDSRLLS-------NT------QPGPGYPPSAPPYGFSPY-

Human_TB5 MLAARLVCLRTLPSRVFHPAF-T-----------------KASPVVKNSIT

Mouse_TB5 MLAARLVCLRTLPSRVFQPTFIT-----------------KASPLVKNSIT

Rat_TBIM5 MLAARLVCLRTLPSRVFQPTFIT-----------------KASPLVKNSIT

Cow_TBIM5 MLAARLVCLRALPSRVFHPAF-T-----------------KASPVVKNSIT

Sheep_TB5 MLAARLVCLRALPSRVFHPAF-T-----------------KASPAVKNSIT

GrizzlyT5 MLAARLLCLRTLPSRVFQPAF-T-----------------KASPIVKNSIT

Nfseal_T5 MLAARFMCLRTLPSRVFQPAF-T-----------------KASPIVKNSIT

CAslionT5 MLAARFMCLRTLPSRVFQPAF-T-----------------KASPIVKNSIT

HIMonkST5 MLAARLVCLRTLPSRVFQPAF-T-----------------KASPIVKNSIT

GhSharkT5 MLVARFACLRNLPSLSLRPGL-T-----------------QGSVAWRNPSQ

betta_TB5 MLVARLTCLRSLPLAGMRPVL-T-----------------HGSPALRAPTL

YlwPrchT5 MLVARLTCLRSLPLAGLRPVL-S-----------------QGSPALRAPTL

Asbass_T5 MLVARLTCLRSLPLAGLRPVL-T-----------------QGSPALRTSTL

Zfish_TB5 MLLCRLSCLRA-------PLGLR-----------------CAVPQGPRALI

Human_TB6 ---------------------------------------------------

Mouse_TB6 ---------------------------------------------------

Rat_TBIM6 ---------------------------------------------------

cow_TBIM6 ---------------------------------------------------

Sheep_TB6 ---------------------------------------------------

GrizzlyT6 ---------------------------------------------------

CAslionT6 ---------------------------------------------------

Nfseal_T6 ---------------------------------------------------

WeddellT6 ---------------------------------------------------

GhSharkT6 ---------------------------------------------------

Betta_TB6 ---------------------------------------------------

YlwPrchT6 ---------------------------------------------------

AsBass_T6 ---------------------------------------------------

GHbreamT6 ---------------------------------------------------

-------------------------------------------------------------

-------------------------------------------------------------

-------------------------------------------------------------

-------------------------------------------------------------

-------------------------------------------------------------

-------------------------------------------------------------

-------------------------------------------------------------

-------------------------------------------------------------

-------------------------------------------------------------

-------------------------------------------------------------

-------------------------------------------------------------

-------------------------------------------------------------

-------------------------------------------------------------

-------------------------------------------------------------

-------------------------------------------------------------

-------------------------------------------------------------

-------------------------------------------------------------

-------------------------------------------------------------

-------------------------------------------------------------

-------------------------------------------------------------

-------------------------------------------------------------

-------------------------------------------------------------

-------------------------------------------------------------

-----------------------------------------MTS--------EKHSQSL--

-------------------------------------------------------------

-----------------------------------------MAD-P-----DPRYPRSS--

-----------------------------------------MAD-T-----DPGYPRSS--

-----------------------------------------MAD-T-----DPRYPRSS--

-----------------------------------------MAD-F-----DPRYPCSS--

-----------------------------------------MAD-L-----DPRYPCSS--

-----------------------------------------MAD-P-----DPRYPCSS--

-----------------------------------------MAD-P-----DPRYPCSS--

-----------------------------------------MAD-P-----DPRYPCSS--

-----------------------------------------MAN-P-----DSRYPCSS--

-----------------------------------------MAD-S-----NPRYPCSS--

-----------------------------------------MEA--------EHYPRSS--

-----------------------------------------MSA--------EKYPRSS--

-----------------------------------------MSS--------ETYPRSS--

-----------------------------------------MSS--------EKYPRSS--

QPSPYGQPGYPHGPSPYPQGGYPQGPYPQGGYPQGPYPQEGYPQGPYPQGGYPQGPYPQ--

QPSPYGQPGYPHGPSPYPQGGYPQG--------------------PYPQGGYPQGPYPQ--

QPSPYGQPGYPHGPGPYPQGGYPQG--------------------PYPQGGYPQGPYPQ--

QPSPYGQPGYPQGPSPYPQGGYPQGPYPPGGYPQGPYPPGGYPQGPYPPGGYPQGPYPQ--

QPSPYGQPGYPQGPSSYPQGGYPQG----------PYPQGGYPQGPYPQGGYPQGPYPQ--

QPSPYGQPGYPQGPSSYPQGGYPQG----------PYPQGGYPQGPYPQGGYPQGPYPQ--

QPSPYGQPGYPQGPSSYPQGGYPQG----------PYPQGGYPQGPYPQGGYPQGPYPQ--

QPSPYGQPGYPQGPNPYPQGGYPQGLYPPGGYPQGPYPPGGYPQGPYPPGGYPQGPYPQ--

QSSPYGQPGYPQGPSSYPQGGYPQG----------PYPQGGYPQGPYPQGGYPQGPYPQ--

PQAGFGQPAFPSA--GYVQPPYPQSAYGQPG-----FPQGSYGE---PPQAFPQGAFQQ--

--AGYGQPGFPQAGPGFAPGPYPQM--------------------PYPQGPYPQGPYPQ--

--AGYGQPGFPQAGPGFAPGPYPQM--------------------PYPQMPYPQGPYPQ--

--AGYGQPGYPQAGPGFAPGPYPQM--------------------PYPQGPYPQGPY----

E--EATSGEGMKAGAF--------PPAPTA---VPLHPSWAYVD--------PSSSSSY--

E--EATSGEGLKAGTF--------PQGPTA---VPLHPSWAYVD--------PSGSSGY--

E--EATSGEGLKAGAF--------PQGPTA---VPLHPSWAYVD--------PSSSSGY--

E--EATSGEGLKAGAF--------PPAPSA---VPLHPSWAYVD--------PNSSSSY--

E--EATSGEGLKAGAF--------PPAPSA---MPLHPSWAYVD--------PNSNSSY--

E--EATSGEGLKAGAF--------PPAPSA---VPLHPSWAYVD--------P-SSSSY--

E--EATSGEGLKAGAF--------PPAPSA---VPLHPSWAYVD--------P-SSSSY--

E--EATSGEGLKAGAF--------PPAPSA---VPLHPSWAYVD--------P-SSSSY--

-------------------------------------------------------SSSY--

----------------------------------------GFTD-VFHLSLCSGRSPSY--

Q--EATAG--------------------------------------------------Y--

E--EATAG--------------------TS---AP-----CYND-V---------------

Q--EATEG--------------------TS---AP-----CYSD-V---------------

E--EATAG--------------------TS---AP-----CYND-V---------------

A--EATAGDKEQGSGSFTYASQPPPMPPPT---MPMHPSWAYVH-P---GPSPGYSNAY--

P--GYPQPGY-------------------G---HPA----GYPQ-PMP-------------

P--AYPQPGY-------------------G---HPA----GYPQ-PVP-------------

P--AYPQPGY-------------------G---HPA----GYPQ-PIP-------------

---AYPQPGY-------------------G---HPA----GYPQ-PMP-------------

---AYPQPGY-------------------G---HPA----GYPQ-PMP-------------

P--AYPQPGY-------------------G---HPA----GYPQ-PMP-------------

P--AYPQPGY-------------------G---HPA----GYPQ-PMP-------------

---AYPQPGY-------------------G---HPA----GYPQ-PMP-------------

P--PPDNMAYPPAGNMAYPPAGNMAYPPAGNMAYPPAGNVGYPP--AGNMGYPPGGMMG--

---SGPQPG--QPSAP--YPPGPNAPLYPG---QPG----GYPS----------------P

---GGPQPG--QPSAP--YPTGPNVPLYPG---QPG----GYP-----------------P

---GGPQPG--QPSAP--YPMGPNTPLYPG---QPG----GYP-----------------P

---GGPQPG--QPSAP--YPAGPNAPMYPG---QPG----GYPQ-GQPGGYPQGQPGGYPP

---EG-QPGYPQPNDP--YRGQPNDP-YRG---QPNDPYRGQPN-D---------------

T-------------------KN-QWLLTP---------SREYAT--KTRIGIRRGRTGQ--

T-------------------KN-QWLVTP---------SREYAT--KTRIRTHRGKTGQ--

T-------------------KN-QWLLTP---------SREYAT--KTRIRTHRGKTGQ--

T-------------------KN-QWLLTP---------SREYAT--KTRIGIRRGKTTQ--

T-------------------KN-QWLLTP---------SREYAT--KTRIGIRRGKTTQ--

T-------------------KN-QWLLTP---------SREYAT--KTRVGIRRGKIGQ--

T-------------------KN-QWLLTP---------SRQYAT--KTRVGIRRGKTGQ--

T-------------------KN-QWLLTP---------SRQYAT--KTRVGIRRGKTGQ--

T-------------------KN-QWLLTP---------SRQYAT--KTRVGIRRGKTGQ--

Q-------------------KTTQWLLQP---------KQGYAS--RPRFSLKRAKSVR--

L-------------------KACPPLVRP---------QQGYSS--KARFGFRRSRTTR--

L-------------------KACPPLLRP---------QQGYSS--KARFGFRRGKTTK--

L-------------------KACPPLLRP---------QQGFSS--KVRFGFRRGRTTR--

I-------------------RSPALMMRPQ--------QQGFSS--RVRLGLRRAKTPK--

-------------------------------------------------------------

-------------------------------------------------------------

-------------------------------------------------------------

-------------------------------------------------------------

-------------------------------------------------------------

-------------------------------------------------------------

-------------------------------------------------------------

-------------------------------------------------------------

-------------------------------------------------------------

-------------------------------------------------------------

-------------------------------------------------------------

-------------------------------------------------------------

-------------------------------------------------------------

-------------------------------------------------------------

---------MAMPSLSACSSIEDDFNYGS--SVASASV-----------------------

---------MAIPSLYACSSIEDDFNYGS--SVASASV-----------------------

---------MAMPSLYACSSIEDDFNYGS--SVASASV-----------------------

---------MAMPSLYACSSIEDDFNYGS--SVASASV-----------------------

-----------MPSLSACSSIEDDFNYGS--SVASASV-----------------------

---------MAMPSLSACSSIEDDFNYGS--SVASASV-----------------------

---------MATPSLSACSSIEDDFNYGS--SVASASV-----------------------

---------MATPSLSACSSIEDDFNYGS--SVASASV-----------------------

---------MATPSLSACSSIEDDFNYGS--SVASASV-----------------------

---------MATPSLSACSSIEDDFNYGS--SVASASV-----------------------

---------MAMPSLSACSSIEDDFNYGS--SVASASV-----------------------

---------MATPSLSACSSIEDDFNYGS--SVASASV-----------------------

-------------------------------------------------------------

---------MAMPSLSACSSIEDDFNYGS--SVASASV-----------------------

---------MAMPSLSACSSIEDDFNYGS--SVASASV-----------------------

---------MAMPSLSACSSIEDDFNYGS--SVASASV-----------------------

---------MAMPSLSACSSIEDDFNYGS--SVASASV-----------------------

---------MAMPSLSARSSIEDDFNYGS--SVASASV-----------------------

-------------------------------------------------------------

-------------------------------------------------------------

-------------------------------------------------------------

-------------------------------------------------------------

-------------------------------------------------------------

--V-EDDFNYG------TN-------------VATANI-----------------------

-------MLPSIPPKHYSIPLDD-FNYSS--SVAASSV-----------------------

--I-EDDFNYG------SS-------------VASATV-----------------------

--I-EDDFNYG------SC-------------VASASV-----------------------

--I-EDDFNYG------SC-------------VASASV-----------------------

--I-EDDFNYG------SC-------------VASASV-----------------------

--I-EDDFNYG------SC-------------VASASV-----------------------

--I-EDDFNYG------SC-------------VASASV-----------------------

--I-EDDFNYG------SC-------------VASASV-----------------------

--I-EDDFNYG------SC-------------VASASV-----------------------

--I-EDDFNYG------SC-------------VASASV-----------------------

--I-EDDFNYG------SC-------------VASASV-----------------------

--I-EDDFNYG------SN-------------VASASV-----------------------

--I-EDDFNYG------TN-------------VATASV-----------------------

--I-EDDFNYG------TN-------------VATASV-----------------------

--I-EDDFNYG------TN-------------VASASV-----------------------

SPFPPNP--YGQPQVFPGQDPDS-PQHGNYQEEGPPSYYD---NQ---D----FPATNWDD

SPFPPNP--YGQPPPF--QDPGS-PQHGNYQEEGPPSYYD---NQ---D----FPAVNWD-

SPFPPNP--YGQPPPF--QDPGS-PQHGNYQEEGPPSYYD---NQ---D----FPSVNWD-

SPFPPNP--YGQPQAFPAQDPGS-PHHGNYHEEGPPSYYD---NQ---D----FPATNWDD

SPFPPNP--YGQPQAFPAQDPGS-PQHGNYHEEGPPSYYD---NQ---D----FPATNWDD

SPFPPNP--YGQPQAFPAQDPDS-PQHGNYHEEGPPSYYD---NQ---D----FPATNWDD

SPFPPNP--YGQPQAFPAQDPGS-PQHGNYHEEGPPSYYD---NQ---D----FPATNWDD

SPFPPNP--YGQPQAFPAQDPGS-PHHGNYHEEGPPSYYD---NQ---D----FPATNWDD

SPFPPNP--YGQPQAFPAQDPGS-PQHGNYHEEGPPSYYD---NQ---D----FPATNWDD

PPMPPPP----------MMHSNVALGSPGYHDDVPPSYYD---NE---E----FSAANFED

GPYPQGPFQQGPAQPGFLGDPTGPATSPSYHGEGPPSYYD---NE---E----FTGSGFED

GPY-----QQGPGQPGFPGDPTVPAGSPGYHGDGPPSYYD---NE---E----FTNSGFED

--------QQGVAQPGFLSDPSA-VGSAGYHGDVPPSYYD---NE---E----FTNSGFED

----DNGFPTG------DH-------------ELFTTF-------------------SWDD

----EGGFPAG------HH-------------EHFTTF-------------------SWDD

----EGGFPAG------HH-------------ELFSTF-------------------SWDD

----ESGFPTG------DH-------------EFFTTF-------------------SWDD

----ESGFPTG------DH-------------EFFTTF-------------------SWDD

----ENGFPAG------DH-------------ELFTTF-------------------SWDD

----ENGFPAG------DH-------------ELFTTF-------------------SWDD

----ENGFPAG------DH-------------ELFTTF-------------------SWDD

----ENGFPAG------DH-------------ELFTTF-------------------SWDD

----GSGSFSG------ES-------------ELFSSF-------------------SWDD

------------------E-------------EMQVQF-------------------AWDD

--------------------------------EMLTEF-------------------TWDD

--------------------------------EMLTEF-------------------TWDD

--------------------------------EMLTEF-------------------TWDD

--AADMSSPFS------DP-------------SSSNSFDGLGS--------------NWED

-PTHPMPMNYG-PGHGYDG--------E--ERAVSDSFGPG-------E---------WDD

-PVHPMPMNYG---HDYNE--------E--ERAGSDSFRPG-------E---------WDD

-PVHPMPMNYG---HDYSE--------E--ERAGSDSFGPG-------E---------WDD

-PVHPMPMNYG-AGHGYDG--------G--ERAVSDTFGSG-------D---------WDD

-PVQPMPMNYG-PGHGYDG--------G--ERAVSDTFGSG-------D---------WDD

-PIHPMPMRYG-PGQGYDG--------E--ERAVSESFGPG-------E---------WDD

-PIHPMPMRYG-PGQGYDG--------E--ERAVSESFGPG-------E---------WDD

-PIHPMPMRYG-PGQGYDG--------E--ERAVSESFGPG-------E---------WDD

---APPAGSPGYPGGGFPIPPTLPLNPSW-PGPGSGPYGSSGSST-HPESE-GFTSSSWST

GPYPGQPYPPGHPGAGYPS-PPMPPVMP--PTIPTDVMSSG-------DDEFAASGSGWDS

GPYQGQPHPAGPPGAGYP--PPMPPVVP--PTIPSDVLSSG-------D-GFAASGSGWDS

GPYSGQPHPGGHPGAGYPSPPPMPPVIP--PTIPSDVLSSG-------D-EFAAHGSGWDS

GPYPGQPHPGGPPGAGYPSRPPMPPVMP--PTMPSDILSSG-------E-EFAASDSSWDS

-PYRGQP-------AGYP--PPAMPVIP--VMPPPGI---G-------DSEGFTTAEGFES

E-LKEAALEPSMEKIFKIDQMGRWFVAGG----AAVGLGALCYYGLGLSNEIGAIEKAVIW

E-LKEAALEPSMEKIFKIDQMGRWFVAGG----AAVGLGALCYYGLGMSNEIGAIEKAVIW

E-LKEAALEPSLEKVFKIDQMGKWFVAGG----AAVGLGALCYYGLGMSNEIGAIEKAVIW

E-LKEAALEPSVEKIFKIDQMGRWFIAGG----AAVGLGALCYYGLGMSNEIGAIEKAVIW

E-LKEAALEPSVEKIFKIDQMGRWFIAGG----AAVGLGALCYYGLGMSNEIGAIEKAVIW

E-LKEAAMEPSVEKIFKIDQMGRWVIAGG----AAVGLGALCYYGLGMSNEIGAIEKAVIW

E-LKEAAMEPSMEKIFKIDQMGRWFIAGG----AAVGLGALCYYGLGMSNEIGAIEKAVIW

E-LKEAAMEPSMEKIFKIDQMGRWFIAGG----AAVGLGALCYYGLGMSNEIGAIEKAVIW

E-LKEAAMEPSVEKIFKIDQMGRWFIAGG----AAVGLGALCYYGLGMSNEIGAIEKAVIW

EDFKDAAYQPAWETAAKMHNTGKLFVAGG----AAVGLGALCYYGLGMSNEIGAIEKAVIW

DQMKDAAFEPATDTAIRIDSMGRIILAGG----AAVGLGALCYYGLGMSNEMGAIEKAVIW

DQLKEAAFEPATDTAIRIDGMGRMVLAGG----AAVGLGALCYYGLGMSNEIGAIEKAVIW

DQLKEAAFEPATDTAIRIDSMGRMILAGG----AVVGLGALCYYGLGMSNDIGAIEKAMIW

EQLQDAALQ-NTHTASRVDSMGRVLLAGG----AAVGLGALCYYGLGMSSEIGAIEKAAIW

-----------------------------------MNIFDRKINF---D----ALLKFSHI

-----------------------------------MNIFDRKINF---D----ALLKFSHI

-----------------------------------MNIFDRKINF---D----ALLKFSHI

-----------------------------------MNIFDRKINF---D----ALFKFSHI

-----------------------------------MNIFDRKINF---D----ALFKFSHI

-----------------------------------MNIFDRKINF---D----ALLKFSHI

-----------------------------------MNIFDRKMNF---D----ALLKFSHI

-----------------------------------MNIFDRKINF---D----ALLKFSHI

-----------------------------------MNIFDRKINF---D----ALLKFSHI

-----------------------------------MNIFDRNTNI-------NALFKFSQI

-----------------------------------MNVFDRNINI---D----AVFKLSQI

-----------------------------------MNVFDRNINF---D----SLFKFSQI

-----------------------------------MNVFDRNINF---D----ALFKFSQI

-----------------------------------MNVFDRNINI---D----ALFKFSQI

-HIRMAFLRKVYGILCLQFLLTTATTAVFLYFDCMRTFIQGSPVLIL---ASMFGSIGL--

-HIRMAFLRKVYGILCLQFLLTTATTAVFLYFDCMRKFIQGSPVLIL---ASMFVSIGL--

-HIRMAFLRKVYGILCLQFLLTTATTAVFLYFDCMRKIIQGSPVLIL---ASMFVSIGL--

-HIRMAFLRKVYGILCLQFLLTTATTAVFLYFDCMRKFIQGSPVLIL---ASMFVSIGL--

-HIRMAFLRKVYGILCLQFLLTTATTAVFLYFDCMRTFIQGSPVLIL---ASMFGSIGL--

-HIRMAFLRKVYGILCLQFLLTTATTAVFLYFDCMRTFIQGSPVLIL---ASMFGSIGL--

-HIRMAFLRKVYGILCLQFLLTTATTAVFLYFDCMRTFIQGSPVLIL---ASMFGSIGL--

-HIRMAFLRKVYGILCLQFLLTTATTAVFLYFDCTRTFIQGSPVLIL---ASMFGSIGL--

-HIRMAFLRKVYGILCLQFLLTTATTAVFLYFDCMRTFIQGSPVLIL---ASMFGSIGL--

-HIRMAFLRKVYGILCLQFLLTTATTAVFLYFDCMRTFIQGSPVLIL---ASMFGSIGL--

-HIRMAFLRKVYGILCLQFLLTTATTAVFLYFDCMRTFIQGSPVLIL---ASMFGSIGL--

-HIRMAFLRKVYGILCLQFLLTTATTAVFLYFDCMRTFIQGSPVLIL---ASMFGSIGL--

----MAFLRKVYGILCLQFLLTTATTAVFLYFDCMRTFIQGSPVLIL---ASMFGSIGL--

-HIRMAFLRKVYGILCLQFLLTTATTAVFLYFDCMRTFIQVSPVLIL---ASMFGSIGL--

-HIRMAFLRKVYGILCLQFLLTTAMTAVFLYFDCMRTFIQGSPVLIL---ASMFGSIGL--

-HIRMAFLRKVYGILCLQFLLTTATTAVFLYFDCMRTFIQGSPVLIL---ASMFGSIGL--

-HIRMAFLRKVYGILCLQFLLTTATTAVFLYFDCMRTFIQGSPVLIL---ASMFGSIGL--

-HIRMAFLRKVYGILCLQFLLTTATTAVFLYFDCMRTFIQGSPVLIL---ASMFGSIGL--

---------------------------------MHRTFIQGSPVLIL---ASMFGSIGL--

----------------------------------------------------MFGSIGL--

----------------------------------------------------MFGSIGL--

----------------------------------------------------MFGSIGL--

----------------------------------------------------MFGSIGL--

-KIRMTFIRRVYTLLSLQIILTISTTALFMFSKHLNAFVQDNSAVVF---VSAFSSFV---

-HIQLAFLRRVYGILLLQLLLTALVCILFLCFPIINTFLCTYPSIMA---ICPIISMVL--

-HIRMAFLRKVYSILSLQVLLTTVTSTVFLYFESVRTFVHESPALIL---LFALGSLG---

-HIRMAFLRKVYSILSLQVLLTTVTSALFLYFQALRTFVHESPALIV---VFALGSLG---

-HIRMAFLRKVYSILSLQVLLTTVTSALFLYFETLRTFVHDSPALIV---VFALGSLG---

-HIRMAFLRKVYSILSLQVLLTTVTLAFFLYFDSIRTFVHESPALIL---VLALGSLG---

-HIRMAFLRKVYSILSLQVLLTTVTSAIFLYFDSIRTFVHESPALIL---VLTLGSLG---

-HIRMAFLRKVYSILSLQVLLTTVTASFFLYFESIRTFVHESPALIL---VFALGSLG---

-HIRMAFLRKVYSILSLQVLLTTVTASFFIYFESIRTFVHESPALIL---VFALGSLG---

-HIRMAFLRKVYSILSLQVLLTTVTASFFIYFESIRTFVHESPALIL---VFALGSLG---

-HIRLAFLRKVYSILSLQVLLTTVTASFFLYFESIRTFVHESPALIL---MFALGSLG---

-HIRMAFLRKVYSILSLQVLLTTVTASFFIYFESIRTFVHESPALIL---VFALGSLG---

-HIRMAFLRKVYTILSLQIILTTVTSAVFMYSDTIKDFIHTSPAFVL---VPALGSLG---

-QIRMDFLRKVYTLLSLQIILTTATSAVFMFSQTIKDFVHSSPAVVL---VSALGSLV---

-QIRMDFLRKVYTLLSLQIILTTATSALFMFSQPIKEFVHASPAVVL---VSALGSLV---

-QIRMDFLRKVYTLLSLQIILTTATSALFMFSHTIKEFVHASPAMVL---VSALGSLV---

KSIRQAFIRKVFLVLTLQLSVTLSTVSVFTFVAEVKGFVRENVWTYY---V----SYA---

KNIRQAFIRKVFLVLTLQLSVTLSTVAIFTFVGEVKGFVRENVWTYY---V----SYA---

KSIRQAFIRKVFLVLTLQLSVTLSTVAIFTFVGEVKGFVRANVWTYY---V----SYA---

KSIRQAFIRKVFLVLTLQLSVTLSTVAVFTFVGEVKGFVRENVWTYY---V----SYA---

KSIRQAFIRKVFLVLTLQLSVTLSTVAVFTFVGKVKGFVRENVWTYY---V----SYA---

KSIRQAFIRKVFLVLTLQLSVTLSTVAVFTFVGKVKGFVRENVWTYY---V----SYA---

KSIRQAFIRKVFLVLTLQLSVTLSTVAVFTFVGKVKGFVRENVWTYY---V----SYA---

KSIRQAFIRKVFLVLTLQLSVTLSTVAVFTFVGEVKGFVRENVWTYY---V----SYA---

KSIRQAFIRKVFLVLTLQLSVTLSTVAVFTFVGKVKGFVRENVWTYY---V----SYA---

KNIRRAFIRKVYMVLTVQLLVTFIFVCICTFVSDAKKFVRRTPWIYY---A----SYG---

KNIRQAFIRKVFMVLTVQLLVTFSFVAVFTFVDDAKYFVRRNPWTYY---V----SYA---

KNIRQAFIRKVFLVLTVQLMVTFSFVAVFTFVDEAKDFVRKNPWTYY---V----SYA---

KSIRQAFIRKVFMVLTVQLMITFSFVAVFTFVNEAKFFVRRNPWTYY---V----SYA---

QKVRRVFVRKVYTILLIQLLVTLAVVALFTFCDPVKDYVQANPGW-------YWASYA---

QKVRRLFIRKVYTILLVQLLVTLAVVALFTFCDVVKDYVQANPGW-------YWASYA---

QKVRQLFIRKVYTILLVQLLVTLAVVALFTFCDVVKDYVQANPGW-------YWASYA---

QKVRRVFIRKVYTILLIQLLVTLGVVALFTFCDPVKDYVQANPGW-------YWASYA---

QKVRRVFIRKVYTILLIQLLVTLGVVALFTFCDPVKDYVQANPGW-------YWASYA---

QKVRRVFIRKVYTILLIQLLVTLGVVALFTFCDPVKDYVQANPGW-------YWASYA---

QKVRRVFIRKVYTILLIQLLVTLGVVALFTFCDPVKDYVQANPGW-------YWASYA---

QKVRRVFIRKVYTILLIQLLVTLGVVALFTFCDPVKDYVQANPGW-------YWASYA---

QKVRRVFIRKVYTILLIQLLVTLGVVALFTFCDPVKDYVQANPGW-------YWASYA---

KNVRRVFIRKVYIILMIQLSLTAGVIALFTFCDPVRSYIQARPGW-------YWASYA---

KAIRRTFIRKVYAILMVQLLVTVAIVGLFTFCTPVRFFIQTHPSM-------YMASYL---

RNIRRVFIRKVYAILMIQLLVTLAIVALFTFCDPVKDYIQSNPGW-------YWASYA---

RNIRRVFIRKVYTILMIQLLVTLAIVSLFTFCDPVKDYIQSNPGW-------YWASYA---

RNIRRVFIRKVYAILMIQLLVTLAIVALFTFCDPVKDYIQTNPGW-------YWASYA---

KNIRRMFIRKVFCILMVQLMVTFSVVSLFTFCEPVRKFVQYNRVFYL---TSYMTFMG---

RKVRHTFIRKVYSIISVQLLITVAIIAIFTFVEPVSAFVRRN----------VAVYYVSYA

RKVRHSFIQKVYCIISVQLLITVAIIAIFTFVEPVGKYVRNN----------VAVYYVSYA

RKVRHTFIQKVYCIISVQLLITVAIIAVFTFVEPVSEYVRSN----------VAVYYVSYA

RKVRHAFIRKVYTIISIQLLVTVGIIAVFTFVKPVGDFVRAN----------MAVYYASYA

RKVRHAFIRKVYTIISIQLLVTVAIIAVFTFVKPVGDFVRAN----------LAIYYASYA

RKVRHTFIRKVYTIISIQLLITVAIIAIFTFVKPVGDFVRKN----------LYVYYVSYA

RKVRHTFIRKVYTIISIQLLITVAIIAIFTFVKPVGEFVRRN----------LYVYYVSYA

RKVRHTFIRKVYTIISIQLLITVAIIAIFTFVKPVGDFVRRN----------LYVYYVSYA

TAVRHAFIRKVYLILTAQLSVTVAIVAAFTFSESVQKFVQGNSALYWTAYGVFIVVYLV--

LSIRHAFIRKVYLILASQLLVTTAIVAIFTFVEPVRLFVQRNQAVYW----ASYAVYA---

LSIRHAFIRKVYLILASQLLVTTGIVAIFTFVHPVKTFVQENRAVYW----ASYAVYI---

LSIRHAFIRKVYLILAAQLLVTTAIVAVFTFVQPVRVFVQRNRAIYW----ASYGVYL---

LSIRHAFIRKVYLILASQLLVTTAIVAVFTFVQPVRYFVQKNQAIYW----ASYAVYI---

TDVRHSFIRKVYLILAAQLLVTAAVVAIFTFVEPVGLFVRKNPAIYW----VSYAIYF---

PQYVKDRIHSTYMYLAGSIGLTALSAIAISRTPVLMNFMMRGSWVTI---G---VTFAA--

PQYVKDRIHSTYMYLAGSIGLTALSALAVARTPALMNFMMTGSWVTI---G---ATFAA--

PQYVKDRIHSTYMYLAGSIGLTALSALALARSPALMNFMMTGSWMTI---G---ATFAA--

PQYVKDRIHSTYMYLAGSIGLTALSAVAVSRTPALMNFMMRGSWITI---G---ATFAA--

PQYVKDRIHSTYMYLAGSIGLTALSAMAVSRTPALMNFMMRGSWITI---G---ATFAA--

PQYVKDRIHSTYMYLAGSIGLTALSALAVSRTPVLMNFMMRGSWVTI---G---ATFAA--

PQYVKDRIHSTYMYLAGSIGLTALSALAVSRTPVLMNFMMRGSWVTI---G---ATFAA--

PQYVKDRIHSTYMYLAGSIGLTALSALAVSRTPVLMNFMMRGSWVTI---G---ATFAA--

PQYVKDRIHSTYMYLAGSIGLTALSALAVSRTPVLMNFMMRGSWVTI---G---ATFAA--

PQYVKERIRSTYMYFAGSIGLTALSAVAVSRSPMLMNLLMKNSFLAI---G---ATFAA--

PQYVKDRIHSTYMYFAGSVGLTALSAVAVSRTPALLGMMMRGSWLAI---G---ATFAA--

PQYVKDRIHSTYMYFAGSIGLTALSAVAVSRTPALMGLMMRGSWLAI---G---ATFAA--

PQYVKDRIHSTYMYFAGSVGLTALSAVAVSRTPALMGLMMRGSWLAI---G---ATFAA--

PQYVRDRIHSTYLYFAGSVGLTALSAVAISRTPVLMGIMMSNSWLAI---G---ATFAA--

TPSTQQHLKKVYASFALCMFVAAAGAYVHMVTHFIQAGLLSALGSLI---L----MIW---

TPSTQQHLKKVYASFALCMFVAAAGAYVHVVTHFIQAGLLSALGSLA---L----MIW---

TPSTQQHLKKVYASFALCMFVAAAGAYVHVVTRFIQAGLLSALGALA---L----MIC---

TPSTQQHLKKVYASFALCMFVAAAGAYIHVVTHFIQAGLLSALGSLG---L----MIW---

TPSTQQHLKKVYASFALCMFVAAAGAYVHVVTHFIQAGLLSALGSLG---L----MIW---

TPSTQQHLKKVYASFALCMFVAAAGAYVHVVTHFLQAGLLSALGSLG---L----MIW---

TPSTQQHLKKVYASFALCMFVAAMGAYVHVVTHFLQAGLLSALGSLG---L----MIW---

TPSTQQHLKKVYASFALCMFVAAMGAYVHVVTHFLQAGLLSALGSLG---L----MIW---

TPSTQQHLKKVYASFALCMFVAAAGAYVHVVTHFLQAGLLSALGSLG---L----MIW---

SPSTQQHLKKVYSSLAICMFVAAAGAYVHVVTRIFQGGLLSMLLSLG--------LMLW--

SHSTQAHLKNVYSSLSVCMILAAAGSYVHVVTRLFQGGILSALGSLG---L----MFW---

SHSTQVHLKNVYSSLAVCMFVAAAGSYVHVVTRLFQGGMLSVLASLG---M----MFW---

SHSTQVHLKNVYSSLAVCMFVAAAGSYVHVVTRLFQGGLLSVLGSLG---M----MFW---

SHSTQAHLKNVYASLAVCMFVAAAGAYVHVVTGLFQGGVLSVLGSLG---M----MFW---

IFALTL--------HRHKHPLNLYLLCGFTLSESLTLASVVTFYDV----HVVMQAFMLTT

IFALTL--------HRHKHPLNLYLLCGFTLSESLTLASVVTFYDV----HVVMQAFMLTT

IFALTL--------HRHKHPLNLYLLCGFTLSESLTLASVVTFYDV----HVVMQAFMLTT

IFALTL--------HRHKHPLNLYLLCGFTLSESLTLASVVTFYDV----HVVMQAFMLTT

IFALTL--------HRHKHPLNLYLLCGFTLSESLTLASVVTFYDV----HVVMQAFMLTT

IFALTL--------HRHKHPLNLYLLCGFTLSESLTLASVVTFYDV----HVVMQAFMLTT

IFALTL--------HRHKHPLNLYLLCGFTLSESLTLASVVTFYDV----HVVMQAFMLTT

IFALTL--------HRHKHPLNLYLLCGFTLSESLTLASVVTFYDV----HVVMQAFMLTT

IFALTL--------HRHKHPLNLYLLCGFTLSESLTLASVVTFYDV----HVVMQAFMLTT

IFALTL--------HRHKHPLNLYLLCGFTLSESLTLASVVTFYDV----HVVMQAFMLTT

IFALTL--------HRHKHPLNLYLLCGFTLSESLTLASVVTFYDV----HVVMQAFMLTT

IFALTL--------HRHKHPLNLYLLCGFTLSESLTLASVVTFYDV----HVVMQAFMLTT

IFALTL--------HRHKYPLNLCTCFVDLHCRNL--------------------------

IFALTL--------HRHKHPLNLYLLCGFTLSESLTLASVVTFYDV----HVVMQAFMLTT

IFALTL--------HRHKHPLNLYLLCGFTLSESLTLASVVTFYDV----HVVMQAFMLTT

IFALTL--------HRHKHPLNLYLLCGFTLSESLTLASVVTFYDV----HVVMQAFMLTT

IFALTL--------HRHKHPLNLYLLCGFTLSESLTLASVVTFYDV----HVVMQAFMLTT

IFALTL--------HRHKHPLNLYLLCGFTLSESLTLASVVTFYDV----HVVMQAFMLTT

IFALTL--------HRHEHPLNLYILCGFTLLESLTLASVVTFYDA----RIVMQAFMLTT

IFALTL--------HRHEHPLNLYILCGFTLLESLTLASVVTFYDA----RIVMQAFMLTT

IFALTL--------HRHKHPLNLYLLCGFTLLESLTLASVVTFYDA----RIVIQAFMLTT

IFALTL--------HRHKHPLNLYLLCGFTLLESLTLASVVTFYDA----RIVIQAFMLTT

IFALTL--------HRHKHPLNLYLLCGFTLLESLTLASVVTFYDA----RIVMQAFMLTT

LMNVL-------VMYRHKRPVNSYLLVAFTFMEAVSVATTLTFYEY----STILQSLFLTC

LFALFL--------NKQNFPANMYLFVGFTLLEAINVAFFASFYNA----YTVIQAAFMTI

LIFAL-------ILNRHKYPLNLYLLFGFTLLEALTVAVVVTFYDV----YIILQAFILTT

LIFAL-------TLHRHTHPLNLYLLFAFTLSESLAVAAVVTFYDV----YLVLQAFIMTT

LIFAL-------TLHRHTHPLNLYLLFAFTLSEALTVATVVTFYDG----HLVLHAFILTA

LILAL-------TVNRHKHPLNLYLLFGFTLLESLTVAFVVTFYDV----YVVLQAFILTC

LILAL-------TVNRHKHPLNLYLLFGFTLLESLTVAFIVTFYDV----YVVLQAFILTC

LILAL-------TVNRHKHPLNLYLLFGFTLFEALTVAFVVTFYDV----YIILQAFILTT

LILAL-------TVNRHKHPLNLYLLFGFTLFEALTVAFVVTFYDV----YIILQAFILTT

LILAL-------TVNRHKHPLNLYLLFGFTLFEALTVAFVVTFYDV----CIILQAFILTT

LVLAL-------TVNRHKHPLNLYLLFGFTLFEALTVAFVVTFYDV----YIILQAFILTT

LILAL-------TVNRHKHPLNLYLLFGFTLFEALTVAFVVTFYDV----YIILQAFILTT

LIIAL-------AIYRHQHPINLYLLFAFTLFEAITVATAVTFYQY----SVVLQAFVLTT

LLLAL-------AMYRHQHPANLYLLFAFTLLEAVSVATALTFYEY----STVLQALFLTC

LLLAL-------AVYRHQHPVNLYLLLVFTLLEAVSVATALTFYEY----STVLQALFLTC

LLLFL-------AIYRHQHPVNFYLLLVFTLLEAVSVATALTFYEY----STVLQALFLTC

VFFISLIVLSCCGDFRRKHPWNLVALSVLTASLSYMVGMIASFYNT----EAVIMAVGITT

IFFISLIVLSCCGDFRRKHPWNLVALSILTVSLSYMVGMIASFYNT----EAVIMAVGITT

IFFISLIVLSCCGDFRRKHPWNLVALSILTISLSYMVGMIASFYNT----EAVIMAVGITT

IFFVSLIVLSCCGDFRRKHPWNLVALSILTVSLSYMVGMIASFYNT----EAVIMAVGITT

VFFISLIVLSCCGDFRRKHPWNLVALSVLTVSLSYMVGMIASFYNT----EAVIMAVGITT

VFFISLIVLSCCGDFRRKHPWNLVALSILTVSLSYMVGMIASFYNT----EAVIMAVGITT

VFFISLIVLSCCGDFRRKHPWNLVALSILTVSLSYMVGMIASFYNT----EAVIMAVGITT

VFFVSLIVLSCCGDFRRKHPWNLVALSILTVSLSYMVGMIASFYNT----EAVIMAVGITT

VFFISLIVLSCCGDFRRKHPWNLIALSILTVSLSYMVGMIASFYNT----EAVIMAVGITT

VFFVTLIALSCCGQIRRKHPWNLIALSILTLSMSYMAGMIASFYST----DSVVMAVGITA

VFFVSLIVLSCCGDFRRKHPWNLVALSILTLSLSYMVGMIASFYNT----DTVIIAVGITA

VFFVSLIVLSCCGDFRRKHPWNLVALSILTLSLSYMVGMIASFYNT----ETVIMAVGITA

VFFVSLIVLSCCGDFRRKHPWNLVALSILTLSLSYMVGMIASFYDT----ETVIMAVGITA

VFFATYLTLACCSGPRRHFPWNLILLTVFTLSMAYLTGMLSSYYNT----TSVLLCLGITA

VFFATYLTLACCSGPRRHFPWNLILLTIFTLSMAYLTGMLSSYYNT----TSVLLCLVITA

VFFATYLTLACCSGPRRHFPWNLILLTIFTLSMAYLTGMLSSYYNT----TSVLLCLGITA

VFFATYLTLACCSGPRRHFPWNLILLTIFTLSMAYLTGMLSSYYNT----TSVLLCLSITA

VFFATYLTLACCSGPRRHFPWNLILLTIFTLSMAYLTGMLSSYYNT----TSVLLCLSITA

VFFATYLTLACCSGPRRHFPWNLILLTIFTLSMAYLTGMLSSYYNT----TSVLLCLGITA

VFFATYLTLACCSGPRRHFPWNLILLTIFTLSMAYLTGMLSSYYNT----TSVLLCLGITA

VFFATYLTLACCSGPRRHFPWNLILLTIFTLSMAYLTGMLSSYYNT----TSVLLCLGITA

VFFATYLTLACCSGPRRHFPWNLILLTIFTLSMAYLTGMLSSYYNT----TSVLLCLGITA

VFFVTYMTLACCPGPRRYFPWNLILLGIFTLAMSYMTGMMSSFYNT----KSVLLCFTITA

MFFATYIALSCCGELRRQFPWNITLLVIFTLSMAFMMGFVSSFYNT----KSVVLCLGITA

VFFVTYLTLSCCSAPRRQFPWNLILLAIFTLSLSYMTGMLSSFYNT----KSVVMCLGITV

VFFVTYLTLSCCSAPRRQFPWNLILLGIFTLSLSYMTGMLSSFYNT----KSVVMCLGITV

VFFVTYLTLSCCSTPRRQFPWNLILLAIFTLSLSYMTGMLSSFYNT----KSVVICLGITA

TYLM----LVCSTNARRRYPTNMILLAIFTLAMSYMAGMLASYHNT----KVVMLSVGITA

VFVVTYLILACCQGPRRRFPWNIILLTLFTFAMGFMTGTISSMYQT----KAVIIAMIITA

VFLVTYLTLACCQGPRRRFPWNIILLTIFTLALGFVTGTISSMYET----KAVIIAMIITA

VFIVTYLILVCCQGPRRRFPWNIILLTIFTLALGFMTGAISSMYET----KAVIIAMIITA

VFLVTYLTLACCQGPRRRFPWNIILLIIFTLAMAYMTGTISSVYKT----KAVIIAMIITA

VFLATYLTLICCQGPRRRFPWNIILLILLTLAMAYMTGTISSVYKT----KAVIIAMIITA

VFLATYLTLACCQGPRRRFPWNIILLTIFTLAMGFMTGTISSVYET----KAVIIAMIITA

VFLATYLTLACCQGPRRRFPWNIILLTIFTLAMGFMTGTISSVYET----KAVIIAMIITA

VFLATYLTLACCQGPRRRFPWNIILLTIFTLAMGFMTGTISSVYET----KVVIIAMIITA

--------LICCEGPRRRFPWNVILLAIFTLAMSYLTGTIASYYST----KSVFMCLGITT

---VTHIVLVCCKGPRRKFPWNIILLFIFTLALSYMSGCISSFYDT----KAVFLALGVTG

---VTHLVLVCCKGPRRKFPWNVILLLIFTLALSYMTGAISSYYST----KAVFLALGITA

---VTHLVLVCCKGPRRKFPWNLILLSIFTLAMSYMTGSISSYYDT----KAVFLAMGITA

---VTHLVLVCCKGPRRKFPWNVILLFLFTLALSYMTGSISSYYDT----KAVFLALGITA

---VTHIVLVCCQGPRRRFPWNLLLLAIFTLALSFMTGNIASYYST----RAVFLALAITV

--MVGAGMLVRSIPYDQSPGPKHLAWLLHSGVMGAVVAPLTILGGP----LLIRAAWYTAG

--MIGAGMLVHSISYEQSPGPKHLAWMLHSGVMGAVVAPLTILGGP----LLLRAAWYTAG

--MIGAGMLVQSISYEQSPGPKHLAWMLHSGVMGAVVAPLTILGGP----LLLRAAWYTAG

--MIGAGMLVQSISYEQSPGPKHLAWLLHSGVMGAVVAPLTILGGP----LLLRAAWYTAG

--MIGAGMLVQSISYEQSPGPKHLAWLLHSGVMGAVVAPLTILGGP----LLVRAAWYTAG

--MIGAGMMVQSIPYDQSPGPKHLAWLLHSGVMGAVVAPLTILGGP----LLVRAAWYTAG

--MIGAGMMVQSIPYDQSPVPKHLAWLLHSGVLGAVVAPLTVLGGP----LLIRAAWYTAG

--MIGAGMMVQSIPYDQSPVPKHLAWLLHSGVLGAAVAPLTVLGGP----LLIRAAWYTAG

--MIGAGMMVRSIPYDQSPVPKHVAWLLHSGVLGAVVAPLTMLGGP----LLIRAAWYTAG

--MIGAGMLVRSLPYTERPSMKHLAWLLHAGVMGAVVAPVTLLGGP----LLIRAAWYTAG

--MIGAGMLVRSISYEHSPVPKHLAWMLHAGVMGAVIAPLTLLGGP----LMIRAAWYTAG

--MIGAGMLVRSISYEQSPMPKHLAWMFHAGVMGAVIAPLTLLGGP----LVMRAAWYTAG

--MIGAGMLVRSISYEHSPLPKHLAWMLHAGVMGAVIAPLTLLGGP----LMMRAAWYTAG

--LIGAGVLVRSVSYEHSPVAKHLSWALYAGVFGAVVAPLTLLGGP----LMLRAVWCTAG

LMAT--------PHSHETEQKRLGLLAGFAFLTGVGLGPALEFCIAVNPSILPTAFMGTAM

LMAT--------PHSHETEQKRLGLLAGFAFLTGVGLGPALELCIAVNPSILPTAFMGTAM

LMAT--------PHSHETEQKRLGLLAGFAFLTGVGLGPALELCIAINPSILPTAFMGTAM

LMAT--------PHSHETEQKRLGLLAGFAFLTGVGLGPALDLCIAINPSILPTAFMGTAM

LMAT--------PHSHETEQKRLGLLAGFAFLTGVGLGPALDLCIAINPSILPTAFMGTAM

LMTV--------PHSHETEQKRLGLLAGFAFLTGVGLGPALELCIAINPSILPTAFMGTAM

LMTT--------PHSHETEQKRLGLLAGFAFLTGVGLGPALELCIAINPSILPTAFMGTAM

LMTT--------PHSHETEQKRLGLLAGFAFLTGVGLGPALELCIAINPSILPTAFMGTAM

LMTT--------PHSHETEQKRLGLLAGFAFLTGVGLGPALELCIAINPSILPTAFMGTAM

LSFT--------PHNSETESKRLLILAGFAFFTGTGLGPIMDFVISVDPSIIPTSFLATAL

LAMT--------PHNPETEKKRLAILAGFAFLTGLGLGPTLDFVIAVNPSIIATAFMGTSV

LAMT--------PHNSKTEKKRLAILAGFAFLTGVGLGPTLDFVIAVNPSIIVTAFMGTSV

LAMT--------PHNPETEKKRLAILAGFAFFTGVGLGPTLDFVIAVNPSIIVTAFMGTSV

LAMT--------PHNPETEKKRLGMLAVFAFLTGVGLGPTLDFVIAVNPSIIMTAFMGTSM

AAFLALTTYTLQSKRDFSKLGAGLFAALWILILSGLLGI----F-VQNETVKLVLSAFGAL

AVFLALTAYTLQSKRDFSKLGAGLFVTLWILILSGLLRI----F-VQSETVELVLSAFGAL

AVFLALTAYTLQSKRDFSKLGAGLFVTLWILILSGLLRI----F-VQSETVELVLSAFGAL

AVFLALTAYTLQSKRDFSKLGAGLFVTLWILILSGLLRI----F-VQSETVELVLSAFGAL

AVFLALTTYTLQSKRDFSKLGAGLFATLWILILSGLLRI----F-VQNETVELVLSALGPL

AVFLALTTYTLQSKRDFSKLGAGLFATLWILILSGLLRI----F-VQNETVELVLSALGPL

AAFLALTTYTLQSKRDFSKLGAGLFAALWILILSGLLRI----F-VQNETVELVMSAFGAL

AAFLALTTYTLQSKRDFSKLGAGLFAALWILILSGLLRI----F-VQNETVELVLSAFGAL

AAFLALTTYTLQSKRDFSKLGAGLFAALWILILSGLLRI----F-VQNETVELALSAFGAL

AAFLALTTYTLQSKRDFSKLGAGLFAALWILILSGLLRI----F-VQNETVELVMSAFGAL

AAFLALTTYTLQSKRDFSKLGAGLFAALWILILSGLLGI----F-VQNETVELVLSAFGAL

AVFLALTTYTLQSKRDFSKLGAGLFAALWILILSGLLRI----F-VQNETVELVLSAFGAL

-------------------------------------------------------------

AVFLALTTYTLQSKRDFSKLGAGLFATLWILILSGLLRI----F-VQNETVELVLSAFGAL

AVFLALTTYTLQSKRDFSKLGAGLFATLWILILSGLLRI----F-VQNETVELVLSAFGAL

AVFLALTTYTLQSKRDFSKLGAGLFATLWILILSGLLRI----F-VQNETVELVLSAFGAL

AVFLALTTYTLQSKRDFSKLGAGLFATLWILILSGLLRI----F-VQNETVELVLSAFGAL

AVFLALTTYTLQSKRDFSKLGAGLFATLWILILSGLLRI----F-VQNETVELVLSAFGAL

AVFLALTTYTLQSKRDFSKLVTGLFAAFWILILSGVLRI----K-FKIELIKNI-------

AVFLALTTYTLQSKRDFSKLVTGLFAAFWILILSGVLRI----K-FKIELIKNI-------

AVFLALTTCTLQSKRDFSKLVTGLFAAFWILILSGVLRI----K-FKIELIKNI-------

AVFLALTTCTLQSKRDFSKLVTGLFADFWILILSGVLRI----K-FKIELIKNI-------

AVFLALTTCTLQSKRDFSKLVTGLFAAFWILILSGVLRI----K-FKIELIKNI-------

AVFAGLTVYTFQSKKDFSKLGAGLFACILIFVIAMLIQL----F-YNSDSADLVISGFGAV

VAFTGLTVYTFQSTRNFSKLGAYLFSCLCVLCVGCILSMV---F--YNEILEIVLAAFGCF

TVFFGLTVYTLQSKKDFSKFGAGLFALLWILCLSGFLKF----F-FYSEIMELVLAAAGAL

AVFLGLTAYTLQSKRDFTKFGAGLFAGLWILCLAGFLKL----F-FYSETMELVLASLGAL

AVFLGLTAYTLQSKRDFSKFGAGLFACLWILCLAGFLKV----F-FYSQTVELVLASLGAL

AVFLGLTVYTLQSKRDFSKFGAGLFAGLWILCLSGILRL----F-FYSETVELVLAAGGAL

AVFLGLTVYTLQSKRDFSKFGAGLFAGLWILCLSGILRL----F-FYSETVELVLAAGGAL

AVFLGLTGYTLQSKRDFSKFGAGLFAVLWILCLSGILKL----F-FYSQTMELVLAAVGAL

AVFLGLTVYTLQSKRDFSKFGAGLFAVLWILCLSGILKL----F-FYSQTMELVLAAVGAL

AVFLGLTVYTLQSKRDFSKFGAGLFAVLWILCLSGILKL----F-FYSQTMELVLAAVGAL

AVFLGLTVYTLQSKRDFSKFGAGLFAVLWILCLSGILKL----F-FYSQTMELILAAVGAL

AVFLGLTVYTLQSKRDFSKFGAGLFAVLWILCLSGILKL----F-FYSQTMELVLAAVGAL

AVFLGLTSYTFQSKRDFSKYGAGLFACLWILILAGFFRL----F-FFSETMELVFASAGAL

AVFAGLTAYTFQSKRDFSKLGAGLFACLWILIIASFMRF----F-FNSDSTELVFAGAGAL

AVFAGLTVYTLQSKRDFSKMGAGLFACLWILLIASFMRL----F-FNSDGTELVMAGAGAL

AVFVGLTAYTFQSKRDFSKMGAGLFACLWILVIASFMRF----F-FNNDSTEVVLAGAGAL

AVCFTVVIFSMQTRYDFTSCMGVLLVSMVVLFIFAILCI----F-IRNRILEIVYASLGAL

AVCFTVVIFSMQTRYDFTSCMGVLLVSVVVLFIFAILCI----F-IRNRILEIVYASLGAL

AVCFTVVIFSMQTRYDFTSCMGVLLVSVVVLFIFAILCI----F-IRNRILEIVYASLGAL

TVCFTVVIFSMQTRYDFTSCVGVLLVSVVVLILFAILCI----F-IRSRVLEIVYASLGAL

TVCFTVVIFSMQTRYDFTSCMGVLLVSLVVLVIFAILCI----F-IRNRILEIVYASLGAL

TVCFTVVIFSMQTRYDFTSCMGVLLVSLVVLVIFAILCI----F-IRNRILEIVYASLGAL

TVCFTVVIFSMQTRYDFTSCMGVLLVSLVVLVIFAILCI----F-IRNRILEIVYASLGAL

TVCFTVVIFSMQTRYDFTSCVGVLLVSVVVLILFAILCI----F-IRSRVLEIVYASLGAL

TVCFTVVIFSMQTRYDFTSCMGVLLVSLVVLVVFAVLCI----F-IRNRILEIVYASLGAL

AVCFTVVIFSMQTKYDFTSCMGVMLVSVVVLFFFGILCI----F-IQNKILQIVYASVGAL

VVCFTVVLFSLQSKYDFTSCRGVLFVCLIVLLLFSILCI----F-IRDKILHIVYASLGAL

VVCFTVVLFSLQSKYDFTSCRGVLFVCLIVLLLFSFLCI----F-IRHKILHIVYASLGAL

VVCFTVVLFSLQSKYDFTSCRGVLFVCLIVLLLFSILCI----F-IRHRILHIVYASLGAL

LVCLSVTVFSFQTKFDFTSCQGVLFVLLMTLFFSGLILAILLPF-QYVPWLHAVYAALGAG

LVCLSVTIFSFQTKFDFTSCQGVLFVLLMTLFFSGLLLAVLLPF-QYVPWLHAVYAVLGAG

LVCLSVTIFSFQTKFDFTSCHGVLFVLLMTLFFSGLLLAILLPF-QYVPWLHAVYAVLGAG

LVCLSVTVFSFQTKFDFTSCQGVLFVLLMTLFFSGLILAILLPF-QYVPWLHAVYAVLGAG

LVCLSVTVFSFQTKFDFTSCQGVLFVLLMTLFFSGLILAILLPF-QYVPWLHAVYAVLGAG

LVCLSVTVFSFQTKFDFTSCQGVIFVLLMTLFFSGLILAILLPF-QYVPWLHAVYAVLGAG

LVCLSVTVFSFQTKFDFTSCQGVIFVLLMTLFFSGLILAILLPF-QYVPWLHAVYAVLGAG

LVCLSVTVFSFQTKFDFTSCQGVIFVLLMTLFFSGLILAILLPF-QYVPWLHAVYAVLGAG

LVCLSVTVFSFQTKFDFTSCQGVIFVLLMTLFFSGLILAILLPF-QYVPWLHAVYAVLGAG

LVCLAVTIFSFQTKFDFTSCHGVLFVLLIVLCISGLVLAIVLPF-QYVPWLHAVYAVLGAI

LVCLSVTVFSFQSKVDVTSCQGILFSLCMVMLLCAITLSIVVPF-GYVPWLHAIYAVLGAI

AVCLLVTIFSFQTKFDVTSYQGVLFVFCMVMFISGLVLALVLPF-QYVPWLDAIYAALGAI

AVCLLVTVFSFQTKFDVTSYQGVLFVFCMVMFVSGLVLALVLPF-QYVPWLDATYAVLGAI

AVCLLVTVFSFQTKFDVTSYQGVLFVFCTVMFISGLVLAFILPF-QYVPWLDGIFAALGAI

LVCLAITLFCFQSRVDFTTCHGLLFSLMMVLMITGLLLFFTAPF-GYIPWLHTAYAGFGAL

VVSISVTIFCFQTKVDFTSCTGLFCVLGIVLLVTGIVTSIVLYF-QYVYWLHMLYAALGAI

VVSISVTIFCFQTKVDFTSCTGLFCVLGIVLMVTGIVTSIVLIF-KYIYWLHMVYAALGAI

VVSISVTIFCFQTKVDFTSCTGLICVLGIVLAVTGAVTSVVLFF-EYIYWLHMVYAGLGAI

VVSISVTIFCFQTKVDFTSCTGLFCVLGIVMTVTGIITVIVLVF-KYVYWLHMVYAALGAI

VVSISVTIFCFQTKVDFTSCTGLFCVLAIVMVVTGIITAIVLAF-KYVYWLHMVYAAVGAI

VVSISVTIFCFQTKVDFTSCTGLFCVLGIVMMVTGIVTAIVLSF-KYIYWLHMVYAALGAI

VVSISVTIFCFQTKVDFTSCTGLFCVLGIVMMVTGIVTAIVLSF-KYIYWLHMVYAALGAI

VVSISVTIFCFQTKVDFTSCTGLFCVLGIVMMVTGIVTAIVLSF-KYIYWLHMVYAALGAI

LVCLGVTLFCFQTKVDFTSCTGLFCALGIGLFVGGIVISIVLSF-KYIPWLHSLYAAAAAV

VVCVAVTVFCFQTKVDFTKCQGLFCVLGIVIFVLGIITAIVLSF-KYIPWLHMLYAALGAI

VVCIAVTVFCFQTKVDFTKCQGLFCVLGIVIFVTGIITTIVLSF-KYILWLHMLYAALGAI

VVCIAVTVFCFQTKVDFTKCQGLFCVLGIVMFVTGIITCIVLSF-KYIPWLHMLYAAMGAI

VVCIAVTVFCFQTKVDFTKCQGLFCVLGIVMLLTGIIATIVLSF-KYILWLHMLYAAMGAI

VVCVAVTVFCFQTKVDFTKCSGFFCVLGIVVFVTGIITAIVLSF-KYVPWLHMLYASIGAI

IVGGLSTVAMCAPSEKFLNMGAPLGVGLGLVFVSSLGSMFLPPTTVAGATLYSVAMYGGLV

IVGGLSTVAMCAPSEKFLNMGAPLGVGLGLVFASSLGSMFLPPTSVAGATLYSVAMYGGLV

IVGGLSTVAMCAPSEKFLNMGAPLGVGLGLVFASSLGSMFLPPTSVAGATLYSVAMYGGLV

IVGGLSTVAMCAPSEKFLNMGAPLGVGLGVVFVSSLGSMFLPPTTVAGATLYSVAVYGGLV

IVGGLSTVAMCAPSEKFLNMGAPLGVGLGVVFVSSLGSMFLPPTTVAGATLYSVAVYGGLV

IVGGLSTVAMCAPSEKFLNMGAPLGVGLGLVFVSSLGSMFLPPTTVAGATLYSVAIYGGLV

IVGGLSAVAVCAPSEKFLNMGAPLGVGLGLVFVSSLGSMFLPPTTVAGATLYSVAIYGGLV

IVGGLSAVAVCAPSEKFLNMGAPLGVGLGLVFVSSLGSMFLPPTTVAGATLYSVAIYGGLV

IVGGLSAVAMCAPSEKFLNMGAPLGVGLGLVFVSSLGSMFLPPTTVAGATLYSVAIYGGLV

IVGGLSTVAMCAPSEKFLNIGGPLAIGLGFVFASSVGSMFLPPTSMLGAGLYSVAIYGGLV

IVGGLSTVAMCAPSEKFLNMGGPLAVGFGVVFASSLGSMFLPPTSAFGAGLYSVAIYGGLV

IVGGLSTVAMCAPSEKFLNMGGPLAVGFGVVFASSIGSMFLPPTSAFGAGLYSVAIYGGLV

IVGGLSTVAVCAPSEKFLNMGGPLAVGFGVVFASSIGSMFLPPTSAFGAGLYSVAVYGGLV

VVGGLSTVAVCAPSERFLNMGGPLAVGLGLVLASTLGSMFLPPSTAVGAGLYSVSVYGGLL

IFTCFTLSALYARRRSYLFLGGILMSALSLLLLSSLGNV----F-FGSIWLFQANLYVGLV

IFTCFSLSALYARRRSYLFLGGILMSAMSLMLLSSLGNL----F-FGSIWLFQANLYLGLL

IFTCFSLSALYARRRSYLFLGGILMSAMSLMFVSSLGNL----F-FGSIWLFQANLYMGLL

IFTCFTLSALYARRRSYLFLGGILMSAMSLMLLSSLGNL----F-FGSVWLFQANLYMGLV

IFTCFTLSALYARRRSYLFLGGILMSAMSLMLLSSLGNL----F-FGSVWLFQANLYMGLV

IFTCFTLSALYARRRSYLFLGGVLMSAMSLMLLSSLGNL----F-FGSIWLFQANLYVGLV

IFTCFTLSALYARRRSYLFLGGVLMSAMSLMLLSSLGNL----F-FGSIWLFQANLYVGLV

IFTCFTLSALYARRRSYLFLGGVLMSAMSLMLLSSLGNL----F-FGSIWLFQANLYVGLV

IFTCFTLSALYARRRSYLFLGGVLMSAMSLMLLSSLGNL----F-FGSIWLFQANLYVGLV

IFSCFTLSALYAQRRSYLFLGGILMSCLTVLCFLPLINLL---F--GSMLLFKVNGRRTFL

IFICFSLSALYAKRRSYLFLGGTLMSGLSFLFLLSVMNI----F-FGSLMLFKAHMYLGLL

IFICFTLSALYAKRRSYLFLGGTLMSGLSLLFLMSVMNM----F-FGSVMVFKAHMYLGLL

IFICFTLSALYAKRRSYLFLGGTLMSGLSILFLMSLMNM----F-FGSLMLFKAHMYLGLL

IFICFSLSALYAKRRSYLFLGGTLMSGLSLLFLMSVMNM----F-FGSVMLFKAHMYLGLL

V----------------FCGFIIYDTHSLIHKLS--------PEEYVLASINLYLDIINLF

V----------------FCGFIIYDTHSLIHKLS--------PEDYVLASINFYLDIINLF

V----------------FCGFIIYDTHSLIHKLS--------PEDYVLASINFYLDIINLF

V----------------FCGFIIYDTHSLIHKLS--------PEDYVLASINFYLDIINLF

-----------------------YSADSL--------------------------------

-----------------------YSADSL--------------------------------

V----------------FCGFIIYDTHSLIHKLS-------------LKSMC---------

V----------------FCGFIIYDTHSLIHKLS--------PEEYVLASINLYLDIINLF

V----------------FCGFIIYDTHSLIHKLS--------PEEYVLASINLYLDIINLF

V----------------FCGFIIYDTHSLIHKLS--------PEEYVLASINLYLDIINLF

V----------------FCGFIIYDTHSLIHKLS--------PEEYVLASINFYLDIINLF

V----------------FCGFIIYDTHSLIHKLS--------PEEYVLASINFYLDIINLF

-------------------------------------------------------------

V----------------FCGFIIYDTHSLIHKLS--------PEEYVLASINFYLDIINLF

V----------------FCGFIIYDTHSLIHKLS--------PEEYVLASINFYLDIINLF

V----------------FCGFIIYDTHSLIHKLS--------PEEYVLASINLYLDIINLF

V----------------FCGFIIYDTHSLIHKLS--------PEEYVLASINFYLDIINLF

V----------------FCGFIIYDTHSLIHKLS--------PEEYVLASINFYLDIINLF

-------------------------------------------------------------

-------------------------------------------------------------

-------------------------------------------------------------

-------------------------------------------------------------

-------------------------------------------------------------

V----------------FCGFIIYDTHKLMKQ--------FSPDEYIVAFIHIYMDIIDLF

L----------------FCGFIIYDTYSLMHRVS--------PEDYLEAVIHLYLDIIILF

L----------------FCGFIIYDTHSLMHK--------LSPEEYVLAAISLYLDIINLF

L----------------FCGFIIYDTHSLMHR--------LSPEEYVIAAISLYMDIINLF

L----------------FCGFIIYDTHSLMHR--------LSPEEYVLAAISLYLDIINLF

L----------------FCGFIIYDTHSLMHR--------LSPEEYVLAAINLYLDIINLF

L----------------FCGFIIYDTHSLMHR--------LSPEEYVLAAINLYLDIINLF

L----------------FCGFIIYDTHSLMHR--------LSPEEYVLAAISLYLDVINLF

L----------------FCGFIIYDTQSLMHR--------LSPEEYVLAAISLYLDVINLF

L----------------FCGFIIYDTHSLMHR--------LSPEEYVLAAISLYLDVINLF

L----------------FCGFIIYDTHSLMHK--------LSPEEYVLAAISLYLDVINLF

L----------------FCGFIIYDTHSLMHR--------LSPEEYVLAAISLYLDVINLF

L----------------FCGFIIYDTHVLMHK--------LSPEEYILASINLYLDIINLF

V----------------FCGFIIYDTHLLMKQ--------LSPEEHILASINLYLDIVNLF

V----------------FCGFIIYDTHLLMKQ--------LSPEEHILASINLYLDIVNLF

I----------------FCGFIIYDTNLLMKQ--------LSPEEHILASINLYLDIVNLF

L----------------FTCFLAVDTQLLLGNKQ----LSLSPEEYVFAALNLYTDIINIF

L----------------FTCFLAVDTQLLLGNKQ----LSLSPEEYVFAALNLYTDIINIF

L----------------FTCFLAVDTQLLLGNKQ----LSLSPEEYVFAALNLYTDIINIF

L----------------FTCFLAVDTQLLLGNKQ----LSLSPEEYVFAALNLYTDIINIF

L----------------FTCFLAVDTQLLLGNKQ----LSLSPEEYVFAALNLYTDIINIF

L----------------FTCFLAVDTQLLLGNKQ----LSLSPEEYVFAALNLYTDIINIF

L----------------FTCFLAVDTQLLLGNKQ----LSLSPEEYVFAALNLYTDIINIF

L----------------FTCFLAVDTQLLLGNKQ----LSLSPEEYVFAALNLYTDIINIF

L----------------FTCFLAVDTQLLLGNKQ----LSLSPEEYVFAALNLYTDIINIF

I----------------FTVVSSWPLPL--------------PQWDIFKL-----------

L----------------FTCFLAVDTQLLLGNKK----LSLSPEEYIFAALNLYTDIINIF

L----------------FTCFLAVDTQLLLGNKK----LALSPEEYIFAALNLYTDIINIF

L----------------FTCFLAVDTQLLLGNKK----MSLSPEEYIFAALNLYTDIINIF

V----------------FTLFLALDTQLLMGNRR----HSLSPEEYIFGALNIYLDIIYIF

V----------------FTLFLAFDTQLLMGNRR----HSLSPEEYIFGALNIYLDIIYIF

V----------------FTLFLAFDTQLLMGNRR----HSLSPEEYIFGALNIYLDIIYIF

V----------------FTLFLAFDTQLLMGSRR----HSLSPEEYIFGALNIYLDIIYIF

V----------------FTLFLAFDTQLLMGSRR----HSLSPEEYIFGALNIYLDIIYIF

V----------------FTLFLAFDTQLLMGNRR----HSLSPEEYIFGALNIYLDIVYIF

V----------------FTLFLAFDTQLLMGNRR----HSLSPEEYIFGALNIYLDIIYIF

V----------------FTLFLAFDTQLLMGNRR----HSLSPEEYIFGALNIYLDIIYIF

V----------------FTLFLAFDTQLLMGNRR----HSLSPEEYIFGALNIYLDIIYIF

I----------------FTMFLAFDTQLLIGNRR----YAISPEEYIFGALNIYLDIIYIF

L----------------FTLFLAFDTQLLLGNKR----YTISPEEYVFATLSIYLDIVYLF

L----------------FTMFLAFDTQLLMGNKR----YTMSPEEYVFATLNIYLDIIYIF

L----------------FTMFLAFDTQLLMGNKR----YTMSPEEYVFATLNLYLDIIYIF

L----------------FTMFLAFDTQLLMGNKR----YTMSPEEYVFATLNIYLDIIYIF

V----------------FTLFLAFDMQLLIGNRR----YSLNPEEHVFGAICLYMDVVYIF

C----------------FTLFLAYDTQLVLGNRK----HTISPEDYITGALQIYTDIIYIF

C----------------FTLFLAYDTQLVLGNRK----HTISPEDYITGALQIYTDIVYIF

C----------------FTLFLAYDTQLVLGNRK----HTISPEDYITGALQIYTDIVYIF

C----------------FTLFLAYDTQMVLGNRK----HTISPEDYITGALQIYTDIVYIF

C----------------FTLFLAYDTQMVLGNRR----HSISPEDYITGALQIYTDIVHIF

C----------------FTLFLAYDTQLVLGNRK----HTISPEDYITGALQIYTDIIYIF

C----------------FTLFLAYDTQLVLGNRK----HTISPEDYITGALQIYTDIIYIF

C----------------FTLFLAYDTQLVLGNRK----HTISPEDYITGALQIYTDIIYIF

I----------------FTLFLAFDTQLILGNRK----HSISPEEYVYAALRLYTDMVQIF

I----------------FTMFLAYHTQLLIGNRK----HSISPEEYVFAALSIYVDIVQIF

V----------------FTMFLAYHTQLLIGNRK----HSISPEEYVFAALSIYVDIIQIF

A----------------FTLFLAYHTQLLIGNRK----HSIGPEEYVFAALSIYVDIVQIF

V----------------FTMFLAYHTQLLIGNRK----YNISPEEYVFAALSIYVDIIQIF

A----------------FTLFLAYHTQLLIGNRK----LSISPEEYVFAALSLYVDIVQIF

L----------------FSMFLLYDTQKVIKRAEVSPMYGVQKYDPINSMLSIYMDTLNIF

L----------------FSMFLLYDTQKVIKRAEITPMYGAQKYDPINSMLTIYMDTLNIF

L----------------FSMFLLYDTQKVVKRAEITPAYGAQKYDPINSMLTIYMDTLNIF

L----------------FSMFLLYDTQKVIKRAEVVPMYGVQKYDPINSMLGIYMDTLNIF

L----------------FSMFLLYDTQKVIKRAEVVPMYGVHKYDPINSMLGIYMDTLNIF

L----------------FSMFLLYDTQKVIKRAEIIPAYGVQKYDPINSMLGIYMDTLNIF

L----------------FSMFLLHDTQKVIKRAEIIPAYGVQKYDPINSMLGIYMDTLNIF

L----------------FSMFLLHDTQKVIKRAEIIPAYGVQKYDPINSMLGIYMDTLNIF

V----------------FSMFLLYDTQKVIKRAEIIPAYGVQKYDPINSMLGIYMDTLNIF

L----------------FGMFLLYDTQKVIKQAESFPVYAVQKFDPINACIGIYMDTLNIF

L----------------FSMFLLYDTQKVIKRAETHPLYGVQKYDPINACMGIYMDTLNIF

L----------------FSMFLLYDTQKVIKRAETHPLYGVQKYDPINACMGIYMDTLNIF

L----------------FSMFLLYDTQKVIKRAETYPVYGVQKYDPINACMGIYMDTLNIF

L----------------FSLFLLYDTQKVIKRAETHPLYSAHKYDPINASLGIFIDTINIF

V----------------MCGFVLFDTQLIIEKAE----H--GDQDYIWHCIDLFLDFITVF

V----------------MCGFVLFDTQLIIEKAE----H--GDKDYIWHCVDLFLDFVTLF

V----------------MCGFVLFDTQLIIEKAE----H--GDKDYIWHCIDLFLDFVTLF

V----------------MCGFVLFDTQLIIEKAE----N--GDKDYIWHCVDLFLDFVTLF

V----------------MCGFVLFDTQLIIEKAE----N--GDKDYIWHCIDLFLDFVTLF

V----------------MCGFVLFDTQLIIEKAE----N--GDKDYIWHCVDLFLDFITLF

V----------------MCGFVLFDTQLIIEKAE----N--GDKDYIWHCVDLFLDFITLF

V----------------MCGFVLFDTQLIIEKAE----N--GDKDYIWHCVDLFLDFITLF

V----------------MCGFVLFDTQLIIEKAE----N--GDKDYIWHCVDLFLDFITLF

TPPPPKKCPAVPCACFPFCCSLTNN------------------------------------

V----------------MCGFVLFDTQLIIEKAE----N--GDKDYVWHCVDLFLDFITIF

I----------------MCGFVLFDTQLIIEKAE----N--GDKDYIWHCVDLFLDFITIF

I----------------MCGFVLFDTQLIIEKAE----N--GDKDYVWHCVDLFLDFVTIF

I----------------MCGFVLFDTQLIIEKAE----N--GDKDYVWHCVDLFLDFITIF

LHLLQLLEVSNKK------

LHLLQLLEVSNKK------

LHLLQLLEVSNKK------

LHLLQLLEVSNKK------

-------SMTRTH------

-------SMTRTH------

-------------------

LHLLQLLEVSNKK------

LHLLQLLEVSNKK------

LHLLQLLEVSNKK------

LHLLQLLEVSNRK------

LHLLQLLEVSNKK------

-------------------

LHLLQLLEVSNKK------

LHLLQLLEVSNKK------

LHLLQLLEVSNKK------

LHLLQLLEVSNKK------

LHLLQLLEVSNKK------

-------------------

-------------------

-------------------

-------------------

-------------------

MRILHILKSIKNQ------

MDFLRILGESKSESKSITV

LHLLRFLEAVNKK------

LHLLKFLEAVNKK------

LHLLKFLDAVNKK------

LHLLRVLEAANKK------

LHLLRVLEAVNKK------

LHLLRFLEAVQKK------

LHLLRFLEAVNKK------

LHLLRFLEAVNKK------

LHLLRFLEAVNKK------

LHLLRFLEAVNKK------

LHILRILESINKK------

LHILRVLDSMKKH------

LHILRLLDSMKKH------

LHILRLLDSMKKH------

LYILTIIGRAKE-------

LYILTIIGRAKE-------

LYILTIIGRAKE-------

LYILTIIGRAKE-------

LYILTIIGRAKE-------

LYILTIIGRAKE-------

LYILTIIGRAKE-------

LYILTIIGRAKE-------

LYILTIIGRAKE-------

-------------------

LYILAIVGRSRE-------

LYILAIVGRSRE-------

LYILTIVGRSRE-------

TFFLQLFGTNRE-------

TFFLQLFGTNRE-------

TFFLQLFGTNRE-------

TFFLQLFGTNRE-------

TFFLQLFGTNRE-------

TFFLQLFGTNRE-------

TFFLQLFGTNRE-------

TFFLQLFGTNRE-------

TFFLQLFGTNRE-------

TFMLQIFGSNRE-------

SFLLQLTGQGRD-------

SFFLQIFGTKQE-------

SFFLQIFGTKQE-------

SFFLQIFGTKRE-------

LFFLQLFGS-RE-------

TFVLQLMGDRN--------

TFVLQLVGSRD--------

TFVLQLVGNRD--------

TFVLQLVGSRD--------

TFVLQLVGRQD--------

TFVLQLVGDRN--------

TFVLQLVGDRN--------

TFMLQLVGDRN--------

INLLQLFGSRE--------

LFLLQIIGASTK-------

LFLLQIIGASTK-------

IFLLQIIGAATK-------

LFLLQIIGASTK-------

IFLLQIIGYAER-------

MRVATMLATGG-NRKK---

MRVATMLATGS-NRKK---

MRVATMLATGS-NRKK---

MRVATILATGG-NRKK---

MRVASILATGG-NRKK---

MRVASILATGS-NRKK---

MRVASILATGG-NRKK---

MRVASILATGG-NRKK---

MRVASILATGS-NRKK---

IRVAAMLGNGGGSSRK---

MRLVMILANGGGSRRK---

MRLVMILANGGGGKRK---

MRLVMILANGGGSRRK---

MRLVMILSGAG-SRRK---

RKLMMILAMNEKDKKKEKK

RKLMLILAFNEKDKKKEKK

RKLMLILAFNEKDKKKEKK

RKLMMILAMNEKDKKKKK-

RKLMMILAMNEKDKKKKK-

RKLMMILAMNEKDKKKEKK

RKLMMILAMNEKDKKKEKK

RKLMMILAMNEKDKKKEKK

RKLMMILAMNEKDKKKEKK

-------------------

RKLMVILALNDKDKKKEKK

RKLMVILAMNDKEKKKEKK

RKLMVILALNDKDKKKEKK

RKLMVILAMNDKDKKKEKK

**Supplementary sequences:**

piggybac_SLP-mCherry-K3L:

actcttcctttttcaatattattgaagcatttatcagggttattgtctcatgagcggatacatatttgaatgtatttagaaaaataaacaaataggggttccgcgcacatttccccgaaaagtgccacctaaattgtaagcgttaatattttgttaaaattcgcgttaaatttttgttaaatcagctcattttttaaccaataggccgaaatcggcaaaatcccttataaatcaaaagaatagaccgagatagggttgagtgttgttccagtttggaacaagagtccactattaaagaacgtggactccaacgtcaaagggcgaaaaaccgtctatcagggcgatggcccactacgtgaaccatcaccctaatcaagttttttggggtcgaggtgccgtaaagcactaaatcggaaccctaaagggagcccccgatttagagcttgacggggaaagccggcgaacgtggcgagaaaggaagggaagaaagcgaaaggagcgggcgctagggcgctggcaagtgtagcggtcacgctgcgcgtaaccaccacacccgccgcgcttaatgcgccgctacagggcgcgtcccattcgccattcaggctgcgcaactgttgggaagggcgatcggtgcgggcctcttcgctattacgccagctggcgaaagggggatgtgctgcaaggcgattaagttgggtaacgccagggttttcccagtcacgacgttgtaaaacgacggccagtgagcgcgcctcgttcattcacgtttttgaacccgtggaggacgggcagactcgcggtgcaaatgtgttttacagcgtgatggagcagatgaagatgctcgacacgctgcagaacacgcagctagattaaccctagaaagataatcatattgtgacgtacgttaaagataatcatgcgtaaaattgacgcatgtgttttatcggtctgtatatcgaggtttatttattaatttgaatagatattaagttttattatatttacacttacatactaataataaattcaacaaacaatttatttatgtttatttatttattaaaaaaaaacaaaaactcaaaatttcttctataaagtaacaaaacttttatgagggacagcccccccccaaagcccccagggatgtaattacgtccctcccccgctagggggcagcagcgagccgcccggggctccgctccggtccggcgctccccccgcatccccgagccggcagcgtgcggggacagcccgggcacggggaaggtggcacgggatcgctttcctctgaacgcttctcgctgctctttgagcctgcagacacctggggggatacggggaaaaGGCCTCCACGGCCAGACTAGAACTTTTAAAAGAAAAGGGGGGATTGGGGGGTACAGTGCAGGGGAAAGAATAGTAGACATAATAGCAACAGACATACAAACTAAAGAATTACAAAAACAAATTACAAAAATTCAAAATTTTATCGATCACGAGACTAGCCTCGAGGGATCatccggtgcccgtcagtgggcagagcgcacatcgcccacagtccccgagaagttggggggaggggtcggcaattgaacgggtgcctagagaaggtggcgcggggtaaactgggaaagtgatgtcgtgtactggctccgcctttttcccgagggtgggggagaaccgtatataagtgcagtagtcgccgtgaacgttctttttcgcaacgggtttgccgccagaacacagctgaagcttcgaggggctcgcatctctccttcacgcgcccgccgccctacctgaggccgccatccacgccggttgagtcgcgttctgccgcctcccgcctgtggtgcctcctgaactgcgtccgccgtctaggtaagtttaaagctcaggtcgagaccgggcctttgtccggcgctcccttggagcctacctagactcagccggctctccacgctttgcctgaccctgcttgctcaactctacgtctttgtttcgttttctgttctgcgccgttacagatccaagctgtgaccggcgccGAATTCCTTCAGGGTGAGTTTGGGGACCCTTGATTGTTCTTTCTTTTTCGCTATTGTAAAATTCATGTTATATGGAGGGGGCAAAGTTTTCAGGGTGTTGTTTAGAATGGGAAGATGTCCCTTGTATCACCATGGACCCTCATGATAATTTTGTTTCTTTCACTTTCTACTCTGTTGACAACCATTGTCTCCTCTTATTTTCTTTTCATTTTCTGTAACTTTTTCGTTAAACTTTAGCTTGCATTTGTAACGAATTTTTAAATTCACTTTTGTTTATTTGTCAGATTGTAAGTACTTTCTCTAATCACTTTTTTTTCAAGGCAATCAGGGTATATTATATTGTACTTCAGCACAGTTTTAGAGAACAATTGTTATAATTAAATGATAAGGTAGAATATTTCTGCATATAAATTCTGGCTGGCGTGGAAATATTCTTATTGGTAGAAACAACTACATCCTGGTCATCATCCTGCCTTTCTCTTTATGGTTACAATGATATACACTGTTTGAGATGAGGATAAAATACTCTGAGTCCAAACCGGGCCCCTCTGCTAACCATGTTCATGCCTTCTTCTTTTTCCTACAGCTCCTGGGCAACGGTACCGGATCCCTGCAGAAGCTTCTAAAAATTGAAATTTTATTTTTTTTTTTTGGAATATAAATGGTGAGCAAGGGCGAGGAGGATAACATGGCCATCATCAAGGAGTTCATGCGCTTCAAGGTGCACATGGAGGGCTCCGTGAACGGCCACGAGTTCGAGATCGAGGGCGAGGGCGAGGGCCGCCCCTACGAGGGCACCCAGACCGCCAAGCTGAAGGTGACCAAGGGTGGCCCCCTGCCCTTCGCCTGGGACATCCTGTCCCCTCAGTTCATGTACGGCTCCAAGGCCTACGTGAAGCACCCCGCCGACATCCCCGACTACTTGAAGCTGTCCTTCCCCGAGGGCTTCAAGTGGGAGCGCGTGATGAACTTCGAGGACGGCGGCGTGGTGACCGTGACCCAGGACTCCTCCCTGCAGGACGGCGAGTTCATCTACAAGGTGAAGCTGCGCGGCACCAACTTCCCCTCCGACGGCCCCGTAATGCAGAAGAAGACCATGGGCTGGGAGGCCTCCTCCGAGCGGATGTACCCCGAGGACGGCGCCCTGAAGGGCGAGATCAAGCAGAGGCTGAAGCTGAAGGACGGCGGCCACTACGACGCTGAGGTCAAGACCACCTACAAGGCCAAGAAGCCCGTGCAGCTGCCCGGCGCCTACAACGTCAACATCAAGTTGGACATCACCTCCCACAACGAGGACTACACCATCGTGGAACAGTACGAACGCGCCGAGGGCCGCCACTCCACCGGCGGCATGGACGAGCTGTACAAGTCTAGATCTGCCACCATGCTTGCATTTTGTTATTCGTTGCCCAATGCGGGTGATGTAATAAAGGGCAGAGTATACGAGAAGGATTATGCTCTATATATTTATCTTTTTGACTATCCTCACTTTGAAGCTATCTTGGCAGAGAGTGTTAAGATGCATATGGATAGATATGTTGAATATAGGGATAAACTGGTAGGGAAAACTGTAAAAGTTAAAGTGATTAGAGTTGATTATACAAAAGGATATATAGATGTCAATTACAAAAGGATGTGTAGACATCAATAATAgCGGCCGCCTAaacttgtttattgcagcttataatggttacaaataaagcaatagcatcacaaatttcacaaataaagcatttttttcactgcattctagttgtggtttgtccaaactcatcaatgtatcttatcatgtctGGATCCggcctccgcgccgggttttggcgcctcccgcgggcgcccccctcctcacggcgagcgctgccacgtcagacgaagggcgcagcgagcgtcctgatccttccgcccggacgctcaggacagcggcccgctgctcataagactcggccttagaaccccagtatcagcagaaggacattttaggacgggacttgggtgactctagggcactggttttctttccagagagcggaacaggcgaggaaaagtagtcccttctcggcgattctgcggagggatctccgtggggcggtgaacgccgatgattatataaggacgcgccgggtgtggcacagctagttccgtcgcagccgggatttgggtcgcggttcttgtttgtggatcgctgtgatcgtcacttggtgagtagcgggctgctgggctggccggggctttcgtggccgccgggccgctcggtgggacggaagcgtgtggagagaccgccaagggctgtagtctgggtccgcgagcaaggttgccctgaactgggggttggggggagcgcagcaaaatggcggctgttcccgagtcttgaatggaagacgcttgtgaggcgggctgtgaggtcgttgaaacaaggtggggggcatggtgggcggcaagaacccaaggtcttgaggccttcgctaatgcgggaaagctcttattcgggtgagatgggctggggcaccatctggggaccctgacgtgaagtttgtcactgactggagaactcggtttgtcgtctgttgcgggggcggcagttatggcggtgccgttgggcagtgcacccgtacctttgggagcgcgcgccctcgtcgtgtcgtgacgtcacccgttctgttggcttataatgcagggtggggccacctgccggtaggtgtgcggtaggcttttctccgtcgcaggacgcagggttcgggcctagggtaggctctcctgaatcgacaggcgccggacctctggtgaggggagggataagtgaggcgtcagtttctttggtcggttttatgtacctatcttcttaagtagctgaagctccggttttgaactatgcgctcggggttggcgagtgtgttttgtgaagttttttaggcaccttttgaaatgtaatcatttgggtcaatatgtaattttcagtgttagactagtaaattgtccgctaaattctggccgtttttggcttttttgttagacgctagcggatAGCGCTTCTCATTGGGCATTCCAGCCTACCCAGCTCGGAGTTAGTTACTCCGTAAGTGTGGCCGGAACAGAGTTCGTCCATCTAAAAAGGGAGGGGACCGGCGAACACATCTAGAGCCACCATGGCCACCGAGTACAAGCCCACGGTGCGCCTCGCCACCCGCGACGACGTCCCCCGGGCCGTACGCACCCTCGCCGCCGCGTTCGCCGACTACCCCGCCACGCGCCACACCGTCGAYCCGGACCGCCACATCGAGCGGGTCACCGAGCTGCAAGAACTCTTCCTCACGCGCGTCGGGCTCGACATCGGCAAGGTGTGGGTCGCGGACGACGGCGCCGCGGTGGCGGTCTGGACCACGCCGGAGAGCGTCGAAGCGGGGGCGGTGTTCGCCGAGATCGGCYCGCGCATGGCCGAGTTGAGCGGTTCCCGGCTGGCCGCGCAGCAACAGATGGAAGGCCTCCTGGCGCCGCACCGGCCCAAGGAGCCCGCGTGGTTCCTGGCCACCGTCGGCGTCTCGCCCGACCACCAGGGCAAGGGTCTGGGCAGCGCCGTCGTGCTCCCCGGAGTGGAGGCGGCCGAGCGCGCYGGGGTGCCCGCCTTCCTGGAGACCTCCGCGCCCCGCAACCTCCCCTTCTACGAGCGGCTCGGCTTCACCGTCACCGCCGACGTCGAGGTGCCCGAAGGACCGCGCACCTGGTGCATGACCCGCAAGCCCGGTGCCGTCGACAATCAACCTCTGGATTACAAAATTTGTGAAAGATTGACTGGTATTCTTAACTATGTTGCTCCTTTTACGCTATGTGGATACGCTGCTTTAATGCCTTTGTATCATGCGTTAACTAACTAaacttgtttattgcagcttataatggttacaaataaagcaatagcatcacaaatttcacaaataaagcatttttttcactgcattctagttgtggtttgtccaaactcatcaatgtatcttatcatgtctggaattgactcaaatgatgtcaattagtctatcagaagctatctggtctcccttccgggggacaagacatccctgtttaatatttaaacagcagtgttcccaaactgggttcttatatcccttgctctggtcaaccaggttgcagggtttcctgtcctcacaggaacgaagtccctaaagaaacagtggcagccaggtttagccccggaattgactggattccttttttagggcccattggtatggctttttccccgtatccccccaggtgtctgcaggctcaaagagcagcgagaagcgttcagaggaaagcgatcccgtgccaccttccccgtgcccgggctgtccccgcacgctgccggctcggggatgcggggggagcgccggaccggagcggagccccgggcggctcgctgctgccccctagcgggggagggacgtaattacatccctgggggctttgggggggggctgtccctgatatctataacaagaaaatatatatataataagttatcacgtaagtagaacatgaaataacaatataattatcgtatgagttaaatcttaaaagtcacgtaaaagataatcatgcgtcattttgactcacgcggtcgttatagttcaaaatcagtgacacttaccgcattgacaagcacgcctcacgggagctccaagcggcgactgagatgtcctaaatgcacagcgacggattcgcgctatttagaaagagagagcaatatttcaagaatgcatgcgtcaattttacgcagactatctttctagggttaatctagctgcatcaggatcatatcgtcgggtcttttttccggctcagtcatcgcccaagctggcgctatctgggcatcggggaggaagaagcccgtgccttttcccgcgaggttgaagcggcatggaaagagtttgccgaggatgactgctgctgcattgacgttgagcgaaaacgcacgtttaccatgatgattcgggaaggtgtggccatgcacgcctttaacggtgaactgttcgttcaggccacctgggataccagttcgtcgcggcttttccggacacagttccggatggtcagcccgaagcgcatcagcaacccgaacaataccggcgacagccggaactgccgtgccggtgtgcagattaatgacagcggtgcggcgctgggatattacgtcagcgaggacgggtatcctggctggatgccgcagaaatggacatggataccccgtgagttacccggcgggcgcgcttggcgtaatcatggtcatagctgtttcctgtgtgaaattgttatccgctcacaattccacacaacatacgagccggaagcataaagtgtaaagcctggggtgcctaatgagtgagctaactcacattaattgcgttgcgctcactgcccgctttccagtcgggaaacctgtcgtgccagctgcattaatgaatcggccaacgcgcggggagaggcggtttgcgtattgggcgctcttccgcttcctcgctcactgactcgctgcgctcggtcgttcggctgcggcgagcggtatcagctcactcaaaggcggtaatacggttatccacagaatcaggggataacgcaggaaagaacatgtgagcaaaaggccagcaaaaggccaggaaccgtaaaaaggccgcgttgctggcgtttttccataggctccgcccccctgacgagcatcacaaaaatcgacgctcaagtcagaggtggcgaaacccgacaggactataaagataccaggcgtttccccctggaagctccctcgtgcgctctcctgttccgaccctgccgcttaccggatacctgtccgcctttctcccttcgggaagcgtggcgctttctcatagctcacgctgtaggtatctcagttcggtgtaggtcgttcgctccaagctgggctgtgtgcacgaaccccccgttcagcccgaccgctgcgccttatccggtaactatcgtcttgagtccaacccggtaagacacgacttatcgccactggcagcagccactggtaacaggattagcagagcgaggtatgtaggcggtgctacagagttcttgaagtggtggcctaactacggctacactagaaggacagtatttggtatctgcgctctgctgaagccagttaccttcggaaaaagagttggtagctcttgatccggcaaacaaaccaccgctggtagcggtggtttttttgtttgcaagcagcagattacgcgcagaaaaaaaggatctcaagaagatcctttgatcttttctacggggtctgacgctcagtggaacgaaaactcacgttaagggattttggtcatgagattatcaaaaaggatcttcacctagatccttttaaattaaaaatgaagttttaaatcaatctaaagtatatatgagtaaacttggtctgacagttaccaatgcttaatcagtgaggcacctatctcagcgatctgtctatttcgttcatccatagttgcctgactccccgtcgtgtagataactacgatacgggagggcttaccatctggccccagtgctgcaatgataccgcgagacccacgctcaccggctccagatttatcagcaataaaccagccagccggaagggccgagcgcagaagtggtcctgcaactttatccgcctccatccagtctattaattgttgccgggaagctagagtaagtagttcgccagttaatagtttgcgcaacgttgttgccattgctacaggcatcgtggtgtcacgctcgtcgtttggtatggcttcattcagctccggttcccaacgatcaaggcgagttacatgatcccccatgttgtgcaaaaaagcggttagctccttcggtcctccgatcgttgtcagaagtaagttggccgcagtgttatcactcatggttatggcagcactgcataattctcttactgtcatgccatccgtaagatgcttttctgtgactggtgagtactcaaccaagtcattctgagaatagtgtatgcggcgaccgagttgctcttgcccggcgtcaatacgggataataccgcgccacatagcagaactttaaaagtgctcatcattggaaaacgttcttcggggcgaaaactctcaaggatcttaccgctgttgagatccagttcgatgtaacccactcgtgcacccaactgatcttcagcatcttttactttcaccagcgtttctgggtgagcaaaaacaggaaggcaaaatgccgcaaaaaagggaataagggcgacacggaaatgttgaatactcat

pBlue_165_mCherry-K3L:

GAGCTCtggcaacctagataaaaaatcaactattgttttatccactttctcgtatgttcgaatgagattataatcctgtattatatgggatgtggaagaattggaacacacgcgtgctatataatgaagagataaatatacactccagtcaagtatttcctttttaaaaaaatccatatataatttatattctgtaacatgttatcccttttcaattaacaatgttagtttataaaaaattaaagaagcgaatcaatgattaatagatgttaagaactataattacgatgtattaataggtatatagttagttggttagttaaaaaagagataacagttactaattaattgttagttattgtctatatgatattacaacctattatttgttctctatagttacattaattaaaattttatatgtgacacccattcatctggagaatacttcttgataccattagtatccatataccatgattctggccaatctgcaaacacagcacGTCGACCCCTGCAGATCGATTTAAAAATTGAAATTTTATTTTTTTTTTTTGGAATATAAATGGTGAGCAAGGGCGAGGAGGATAACATGGCCATCATCAAGGAGTTCATGCGCTTCAAGGTGCACATGGAGGGCTCCGTGAACGGCCACGAGTTCGAGATCGAGGGCGAGGGCGAGGGCCGCCCCTACGAGGGCACCCAGACCGCCAAGCTGAAGGTGACCAAGGGTGGCCCCCTGCCCTTCGCCTGGGACATCCTGTCCCCTCAGTTCATGTACGGCTCCAAGGCCTACGTGAAGCACCCCGCCGACATCCCCGACTACTTGAAGCTGTCCTTCCCCGAGGGCTTCAAGTGGGAGCGCGTGATGAACTTCGAGGACGGCGGCGTGGTGACCGTGACCCAGGACTCCTCCCTGCAGGACGGCGAGTTCATCTACAAGGTGAAGCTGCGCGGCACCAACTTCCCCTCCGACGGCCCCGTAATGCAGAAGAAGACCATGGGCTGGGAGGCCTCCTCCGAGCGGATGTACCCCGAGGACGGCGCCCTGAAGGGCGAGATCAAGCAGAGGCTGAAGCTGAAGGACGGCGGCCACTACGACGCTGAGGTCAAGACCACCTACAAGGCCAAGAAGCCCGTGCAGCTGCCCGGCGCCTACAACGTCAACATCAAGTTGGACATCACCTCCCACAACGAGGACTACACCATCGTGGAACAGTACGAACGCGCCGAGGGCCGCCACTCCACCGGCGGCATGGACGAGCTGTACAAGTCTAGATCTGCCACCATGCTTGCATTTTGTTATTCGTTGCCCAATGCGGGcGATGTAATAAAGGGCAGAGTATACGAGAAGGATTATGCTCTATAcATTTATCTTTTTGACTATCCTCACTcTGAAGCTATCTTGGCAGAGAGTGTTAAGATGCATATGGATAGATATGTTGAATATAGGGATAAACTGGTAGGGAAAACTGTAAAAGTTAAAGTGATTAGAGTTGATTATACAAAAGGATATATAGATGTCAATTACAAAAGGATGTGTAGACATCAATAAATCGATCTAAAAATTGAAATTTTATTTTTTTTTTTTGGAATATAAatgattgaacaagatggattgcacgcaggttctccggccgcttgggtggagaggctattcggctatgactgggcacaacagacaatcggctgctctgatgccgccgtgttccggctgtcagcgcaggggcgcccggttctttttgtcaagaccgacctgtccggtgccctgaatgaactgcaggacgaggcagcgcggctatcgtggctggccacgacgggcgttccttgcgcagctgtgctcgacgttgtcactgaagcgggaagggactggctgctattgggcgaagtgccggggcaggatctcctgtcatctcaccttgctcctgccgagaaagtatccatcatggctgatgcaatgcggcggctgcatacgcttgatccggctacctgcccattcgaccaccaagcgaaacatcgcatcgagcgagcacgtactcggatggaagccggtcttgtcgatcaggatgatctggacgaagagcatcaggggctcgcgccagccgaactgttcgccaggctcaaggcgcgcatgcccgacggcgaggatctcgtcgtgacccatggcgatgcctgcttgccgaatatcatggtggaaaatggccgcttttctggattcatcgactgtggccggctgggtgtggcggaccgctatcaggacatagcgttggctacccgtgatattgctgaagagcttggcggcgaatgggctgaccgcttcctcgtgctttacggtatcgccgctcccgattcgcagcgcatcgccttctatcgccttcttgacgagttcttctgaGCGGGACTCTGGGGTTCGCGAAATGATCGACCAAGCGACGCCCAACCTGCCATCACGAGATTTCGATTCCACCGCCGCCTTCTATGAAAGGTTGGGCTTCGGAATCGTTTTCCGGGACGCCGGCTGGATGATCCTCCAGCGCGGGGATCTCATGCTGGAGTTCTTCGCCCACCCCAACTTGTTTATTGCAGCTTATAATGGTTACAAATAAAGCAATAGCATCACAAATTTCACAAATAAAGCATTTTTTTCACTGCATTCTAGTTGTGGTATCGATaacagcatctttccactttgatagcgcacacgtatgtcgaggtagcctcatccccaggtttatataccttgatgaatcgacacgcgtacttgatgtcctctttcttctcacagaaatgcacaacacaaagtcttttgatatgcttttctatatcattctctaccagtctgaggtagtcgtaacccaccgtgaatccaaatactttgtgtgtattattatcaactccaatgaacaaatatccaccctttgtgttggtaaaagatgagagtataaaggaagctgctgccgtatacgtgttcttagttgcttagctgaaacagatgtatgtttaacaattaatagaattaccaaatttgactaatttaccagcctgaagttctgatctattgaagaactcctctaccaatctctcaattgattcagtgtcttccactccatctggatattcaaattcctgcatttctggtctgggactccatccacctgattccttcagttcgctGCTAGCCATATGGGTACC

**Isolate sequences:**

Isolate 1:

caagtcccataccgcaaatggaatatccattagatttcctagatttactaggataagagaggataaaacgtggaaagaatctactcatctaaacgatttagtaaacttgactaaatcttaatagttacatacaaactgaaaattaaaataacaccatttagttggtggtcgccatggatggtgttattgtatactgtctaaacgcgttagtaaaacatggcgaggaaataaatcatataaaaaatgatttcatgattaaaccatgttgtgaaagagtttgtgaaaaagttaagaacgttcacattggcggacaatctaaaaacaatacagtgattgcagatttgccatatatggataatgctgtatcggatgtatgcaattcactgtataaaaagaatgtatcaagaatatccagatttgctaatttgataaagatagatgacgatgacaagactcctactggtgtatataattattttaaacctaaagatgttattcctgttatcatatctataggaaaggataaagatgtctgtgaactattaatctcatcagacatatcgtgtgcatgcgtggagttaaattcatataaagtagccattcttcccatggatgtttccttttttaccaaaggaaatgcatcattgattattctcctgtttgatttctctatcgatgcagcacctctcttaagaagtgtaaccgataGctttttcgcaacgggtttgccgccagaacacagctgaagcttcgaggggctcgcatctctccttcacgcgcccgccgccctacctgaggccgccatccacgccggttgagtcgcgttctgccgcctcccgcctgtggtgcctcctgaactgcgtccgccgtctagCTCCTGGGCAACGGTACCGGATCCCTGCAGAAGCTTCTAAAAATTGAAATTTTATTTTTTTTTTTTGGAATATAAATGGTGAGCAAGGGCGAGGAGGATAACATGGCCATCATCAAGGAGTTCATGCGCTTCAAGGTGCACATGGAGGGCTCCGTGAACGGCCACGAGTTCGAGATCGAGGGCGAGGGCGAGGGCCGCCCCTACGAGGGCACCCAGACCGCCAAGCTGAAGGTGACCAAGGGTGGCCCCCTGCCCTTCGCCTGGGACATCCTGTCCCCTCAGTTCATGTACGGCTCCAAGGCCTACGTGAAGCACCCCGCCGACATCCCCGACTACTTGAAGCTGTCCTTCCCCGAGGGCTTCAAGTGGGAGCGCGTGATGAACTTCGAGGACGGCGGCGTGGTGACCGTGACCCAGGACTCCTCCCTGCAGGACGGCGAGTTCATCTACAAGGTGAAGCTGCGCGGCACCAACTTCCCCTCCGACGGCCCCGTAATGCAGAAGAAGACCATGGGCTGGGAGGCCTCCTCCGAGCGGATGTACCCCGAGGACGGCGCCCTGAAGGGCGAGATCAAGCAGAGGCTGAAGCTGAAGGACGGCGGCCACTACGACGCTGAGGTCAAGACCACCTACAAGGCCAAGAAGCCCGTGCAGCTGCCCGGCGCCTACAACGTCAACATCAAGTTGGACATCACCTCCCACAACGAGGACTACACCATCGTGGAACAGTACGAACGCGCCGAGGGCCGCCACTCCACCGGCGGCATGGACGAGCTGTACAAGTCTAGATCTGCCACCATGCTTGCATTTTGTTATTCGTTGCCCAATGCGGGcGATGTAATAAAGGGCAGAGTATACGAGAAGGATTATGCTCTATAcATTTATCTTTTTGACTATCCTCACTcTGAAGCTATCTTGGCAGAGAGTGTTAAGATGCATATGGATAGATATGTTGAATATAGGGATAAACTGGTAGGGAAAACTGTAAAAGTTAAAGTGATTAGAGTTGATTATACAAAAGGATATATAGATGTCAATTACAAAAGGATGTGTAGACATCAATAATAgGCGGCCGCCTAaacttgtttattgcagcttataatggttacaaataaagcaatagcatcacaaaAAAAAAAAAAAAAAAAAAAAAAAAAAAAAAAAAAAAAAAAAAAAAAAgaagtgtaaccgataataatgttattatatctagacaccagcgtctacatgacgagcttccgagttccaattggttcaagttttacataagtataaagtccgactattgttctatattatatatggttgttgatggatctgtgatgatgcgatagctgataatagaactcacgcaattattagcaaaaatatattagacaatactacgattaacgatgagtgtagatgctgttattttgaaccacagattaggattcttgatagagatgagatgctcaatggatcatcgtgtgatatgaacagacattgtattatgatgaatttacctgatgtaggcgaatttggatctagtatgttggggaaatatgaacctgacatgattaagattgctctttcggtggctggtaatttaataagaaatcgagactacattcccgggagacgaggatatagctactacgtttacggtatagcctctagataa

Isolate 2:

atgattgcgttattgatactatcgttaacgtgttcagtgtctacctatcgtctgcaaggatttaccaatgccggtatagtagcgtataaaaatattcaagatgataatattgtcttctcaccgtttggttattcgttttctatgtttatgtcgctattgcctgcatcaggtaatactagaatagaattattgaagactatggatttgagaaaaagagatctgggtccagcatttacagaattaatatcaggattagctaagctgaaaacatctaaatatacgtacactgatctaacttatcaaagtttcgtagataatactgtgtgcattaaaccgttgtattatcaacaatatcatagattcggcctatatagattaaactttagacgagatgcggttaataaaattaattctatagtagaacgtagatccggtatgtctaatgtagtagattctaatatgctcgacaataatactctatgggcaatcattaatactatatattttaaaggtacatggcaatatccgtttgatatcactaaaacacgcaatgctagttttactaataagtacggtacgaaaacggttcccatgatgaacgtagttactaaattgcaaggaaatacaatcacaatcgatgacgaagaatatgatatggtgcgccttccgtataaggatgctaatattagtatgtacctggcaataggtgataatatgacccatttcacagattctattacggctgcaaaattagactattggtcgtttcaattagggaataaagtgtacaatcttaaactccctaaattttctatcgaaaataagagggatattaagtcgatagccgaaatgatggctcctagtatgtttaatccagataatgcgtcgtttaaacatatgactagggacccattatatatttataaaatgtttcagaatgcaaagatagatgtcgacgaacaaggaactgtagcagaggcatctactatcatggtagctacggcgagatcatctcctgaaaaactggaatttaatacaccatttgtgttcatcattagacatgatattactggatttatattgtttatgggtaaggtagaatctccttaatattgtttatggatacggtggaaggaatcattattttatttatattgatgggtacgtgaaatctgaattttcttaataaatattatttttattaaatgtgtatatgttgttttgcgatagccatgtatctactaatcagatctattagagatattattaattctggtgcaatatgacaaaattataaaaaatgaaaaaatatacactaattagcgtctcgtttcagacatggatctgtcacgaattaatacttggaagtctaagcagctgaaaagctttctctctagtaaagatacatttaaggcggatgtccatggacatagtgccttgtattatgcaatagctgataataacgtgcgtctagtatgtacgttgttgaacgctggagcattgaaaaatcttctagagaatgaatttccattacatcaggcagccacattagaagataccaaaatagctttttcgcaacgggtttgccgccagaacacagctgaagcttcgaggggctcgcatctctccttcacgcgcccgccgccctacctgaggccgccatccacgccggttgagtcgcgttctgccgcctcccgcctgtggtgcctcctgaactgcgtccgccgtctagCTCCTGGGCAACGGTACCGGATCCCTGCAGAAGCTTCTAAAAATTGAAATTTTATTTTTTTTTTTTGGAATATAAATGGTGAGCAAGGGCGAGGAGGATAACATGGCCATCATCAAGGAGTTCATGCGCTTCAAGGTGCACATGGAGGGCTCCGTGAACGGCCACGAGTTCGAGATCGAGGGCGAGGGCGAGGGCCGCCCCTACGAGGGCACCCAGACCGCCAAGCTGAAGGTGACCAAGGGTGGCCCCCTGCCCTTCGCCTGGGACATCCTGTCCCCTCAGTTCATGTACGGCTCCAAGGCCTACGTGAAGCACCCCGCCGACATCCCCGACTACTTGAAGCTGTCCTTCCCCGAGGGCTTCAAGTGGGAGCGCGTGATGAACTTCGAGGACGGCGGCGTGGTGACCGTGACCCAGGACTCCTCCCTGCAGGACGGCGAGTTCATCTACAAGGTGAAGCTGCGCGGCACCAACTTCCCCTCCGACGGCCCCGTAATGCAGAAGAAGACCATGGGCTGGGAGGCCTCCTCCGAGCGGATGTACCCCGAGGACGGCGCCCTGAAGGGCGAGATCAAGCAGAGGCTGAAGCTGAAGGACGGCGGCCACTACGACGCTGAGGTCAAGACCACCTACAAGGCCAAGAAGCCCGTGCAGCTGCCCGGCGCCTACAACGTCAACATCAAGTTGGACATCACCTCCCACAACGAGGACTACACCATCGTGGAACAGTACGAACGCGCCGAGGGCCGCCACTCCACCGGCGGCATGGACGAGCTGTACAAGTCTAGATCTGCCACCATGCTTGCATTTTGTTATTCGTTGCCCAATGCGGGcGATGTAATAAAGGGCAGAGTATACGAGAAGGATTATGCTCTATAcATTTATCTTTTTGACTATCCTCACTcTGAAGCTATCTTGGCAGAGAGTGTTAAGATGCATATGGATAGATATGTTGAATATAGGGATAAACTGGTAGGGAAAACTGTAAAAGTTAAAGTGATTAGAGTTGATTATACAAAAGGATATATAGATGTCAATTACAAAAGGATGTGTAGACATCAATAATAgGCGGCCGCCTAaacttgtttattgcagcttataatggttacaaataaagcaatagcatcacaaaAAAAAAAAAAAAAAAAAAAAAAAAAAAAAAAAAAAAAAAAAAAAAagaagataccaaaatagtaaagattttgctattcagtggaatggatgattcacaatttgatgacaaaggaaacaccgcattgtattatgcggttgatagtggtaacatgcaaacggtgaaactgtttgttaagaaaaattggagactgatgttctatgggaaaactggatggaaaacttcattttatcatgccgtcatgcttaatgatgtaagtattgtatcatactttctttcagaaataccatctacttttgatctggctattctccttagttgtattcacaccactataaaaaatggacacgtggatatgatgattctcttgctcgactatatgacgtcgacaaacaccaataattcccttctcttcattccggacattaaattggctatagataataaagacattgagatgttacaggctctgttcaaatacgacattaatatctactctgttaatctggaaaatgtactattggatgatgccgaaataactaagatgattatagaaaagcatgttgaatacaagtctgactcctatacaaaagatctcgatatcgtcaagaataataaattggatgaaataattagcaaaaacaaggaactcagactcatgtacgtcaattgtgtaaagaaaaactaa

Isolate 3:

atgaacaaacctaagacagattatgctggttatgcttgctgcgtaatatgcggtctaattgtcggaattatttttacagcgacactattaaaagttgtagaacgtaaattagttcatacaccatcaatagataaaacgataaaagatgcatatattagagaagattgtcctactgactggataagctataataataaatgtatccatttatctactgatcgaaaaacctgggaggaaggacgtaatacatgcaaagctctaaatccaaattcggatctaattaagatagagactccaaacgagttaagttttttaagaagccttagacgaggctattgggtaggagaatccgaaatattaaaccagacaaccccatataattttatagctaaaaatgccacgaagaatggaaatatatttgtagcacaacgaatactcccaaactgcattcgtgttacactatataacaattacactacatttttatcataccactacttcggttagatgttttagaaaaaaataaatatcgcGctttttcgcaacgggtttgccgccagaacacagctgaagcttcgaggggctcgcatctctccttcacgcgcccgccgccctacctgaggccgccatccacgccggttgagtcgcgttctgccgcctcccgcctgtggtgcctcctgaactgcgtccgccgtctagCTCCTGGGCAACGGTACCGGATCCCTGCAGAAGCTTCTAAAAATTGAAATTTTATTTTTTTTTTTTGGAATATAAATGGTGAGCAAGGGCGAGGAGGATAACATGGCCATCATCAAGGAGTTCATGCGCTTCAAGGTGCACATGGAGGGCTCCGTGAACGGCCACGAGTTCGAGATCGAGGGCGAGGGCGAGGGCCGCCCCTACGAGGGCACCCAGACCGCCAAGCTGAAGGTGACCAAGGGTGGCCCCCTGCCCTTCGCCTGGGACATCCTGTCCCCTCAGTTCATGTACGGCTCCAAGGCCTACGTGAAGCACCCCGCCGACATCCCCGACTACTTGAAGCTGTCCTTCCCCGAGGGCTTCAAGTGGGAGCGCGTGATGAACTTCGAGGACGGCGGCGTGGTGACCGTGACCCAGGACTCCTCCCTGCAGGACGGCGAGTTCATCTACAAGGTGAAGCTGCGCGGCACCAACTTCCCCTCCGACGGCCCCGTAATGCAGAAGAAGACCATGGGCTGGGAGGCCTCCTCCGAGCGGATGTACCCCGAGGACGGCGCCCTGAAGGGCGAGATCAAGCAGAGGCTGAAGCTGAAGGACGGCGGCCACTACGACGCTGAGGTCAAGACCACCTACAAGGCCAAGAAGCCCGTGCAGCTGCCCGGCGCCTACAACGTCAACATCAAGTTGGACATCACCTCCCACAACGAGGACTACACCATCGTGGAACAGTACGAACGCGCCGAGGGCCGCCACTCCACCGGCGGCATGGACGAGCTGTACAAGTCTAGATCTGCCACCATGCTTGCATTTTGTTATTCGTTGCCCAATGCGGGcGATGTAATAAAGGGCAGAGTATACGAGAAGGATTATGCTCTATAcATTTATCTTTTTGACTATCCTCACTcTGAAGCTATCTTGGCAGAGAGTGTTAAGATGCATATGGATAGATATGTTGAATATAGGGATAAACTGGTAGGGAAAACTGTAAAAGTTAAAGTGATTAGAGTTGATTATACAAAAGGATATATAGATGTCAATTACAAAAGGATGTGTAGACATCAATAATAgGCGGCCGCCTAaacttgtttattgcagcttataatggttacaaataaagcaatagcatcacaaaAAAAAAAAAAAAAAAAAAAAAAAAAAAAAAAAAAAAAAAAAAAAAagaaaaaaataaatatcgccgtaccgttcttgtttttataaaaataacaattaacaattatcaaattttttctttaatattttacgtggttgaccattcttggtggtaaaataatctcttagtgttggaatggaatgctgtttaatgtttccgcactcatcgtatattttgacgtatgcagtcacatcgtttacgcaatagtcagactgtagttctatcatgcttcctacatcagaaggaggaacagttttaaagtctcttggttttaatctattgccattagttttcatgaaatcctttgttttatccacttcacattttaaataaatgtccactatacattcttctgttaattttactagatcgtcatgggtcatagaatttataggttccgtagtccatggatccaaactagcaaacttcgcgtatacggtatcgcgattagtgtatacaccaacagtatgaaaattaagaaaacagtttaatagatcaacagaaatatttaatcctccgtttgatacagatgcgccatatttatggatttcggattcacacgttgtttgtctgaggggttcgtctagcgttgcttctacgtaaacttcgattcccatatattctttattgtcagaatcgcataccgatttatcatcatacactgtttgaaaactaaatggtatacacatcaaaataataaataataacgagtacat

Isolate 4:

gagaaagagaaagagatagttagtctagatatttttcttagtacaaaagtcaatgttttaaaatatatggacaagaatttgtctgtataaaaacttgtgtgaaattttgtaccaaagaaaaaatgtgagcagtatcccctacatggattttactagatcatttatataccaaaaaatattatacgatctacgttttattatatgattttaacgtgtaaattataaacattattttatgatatacaattgtctggtaacctagatgggcataggggatgttgataagctcgacgagtatatgttgttggacgttattgtttaagaaatagttgatgcatcagaaagagaataaaaaatattttagtgagaccatcgaagagagaaagagataaaacttttttacgactccatcagaaagaggtttaatatttttgtgagaccatcgaagagagaaagagaataaaaatattttatgactccattgaagagagaaagagaaaatgagaatgagaataaaaatattttagtgacaccatcagaaagaggtttaatatttttgtgagaccatcgaagagagaaagagaataaaaatattttatgactccattgaagagagaaagagaaaatgagaatgagaataaaaatattttagtgacaccatcagaaagaggtttaatattttttatgagaccatcaaagagagaaagagaataaaaatatttttgtaaaactttttttatgagaccatcaaagagagaaagaGAATAAAAATATTTTTGTAAAACTTTTTTTGTGAGACCATCAAAGAGAGAAAGAGAATAAAAATATTTTTGTAAAACTTTTTTTATGAGACCATCAAAGAGAGAAAGAGAATAAAAATATTTTTGTAAAACTTTTTTTATGAGACCATCAAAGAGAGAAAGAGAATAAAAATATTTTTGTAAAACTTTTTTTATGAGACCATCAAAGAGAGAAAGAGAATAAAAATATTTTTGTAAAACTTTTTTTATGAGACCATCAAAGAGAGAAAGAGAATAAAAATATTTTTGTAAAACTTTTTTTATGAGACCATCAGAAAGAGGTTTAATATTTTTGTGATACCCTGAAAGGAAATAGGAATAGGAATAGGAATAGTGTCATAATCGTATCACACTATTGAGACAGAAAAAGAAGAAGTAACGAGAGGTAACTTTTTGTGAATGTAGTTAAGAACATTTTTGTTTTGCAAACCGGAATATAGTGTCCGGTACACTTTTAGACCATCGAAGAGAGAAAGAGAATAAAAATATTTTATGACTCCATTGAAGAGAGAAAGAGAAAATGAGAATGAGAATaaaaatattttagtctttttcgcaacgggtttgccgccagaacacagctgaagcttcgaggggctcgcatctctccttcacgcgcccgccgccctacctgaggccgccatccacgccggttgagtcgcgttctgccgcctcccgcctgtggtgcctcctgaactgcgtccgccgtctagCTCCTGGGCAACGGTACCGGATCCCTGCAGAAGCTTCTAAAAATTGAAATTTTATTTTTTTTTTTTGGAATATAAATGGTGAGCAAGGGCGAGGAGGATAACATGGCCATCATCAAGGAGTTCATGCGCTTCAAGGTGCACATGGAGGGCTCCGTGAACGGCCACGAGTTCGAGATCGAGGGCGAGGGCGAGGGCCGCCCCTACGAGGGCACCCAGACCGCCAAGCTGAAGGTGACCAAGGGTGGCCCCCTGCCCTTCGCCTGGGACATCCTGTCCCCTCAGTTCATGTACGGCTCCAAGGCCTACGTGAAGCACCCCGCCGACATCCCCGACTACTTGAAGCTGTCCTTCCCCGAGGGCTTCAAGTGGGAGCGCGTGATGAACTTCGAGGACGGCGGCGTGGTGACCGTGACCCAGGACTCCTCCCTGCAGGACGGCGAGTTCATCTACAAGGTGAAGCTGCGCGGCACCAACTTCCCCTCCGACGGCCCCGTAATGCAGAAGAAGACCATGGGCTGGGAGGCCTCCTCCGAGCGGATGTACCCCGAGGACGGCGCCCTGAAGGGCGAGATCAAGCAGAGGCTGAAGCTGAAGGACGGCGGCCACTACGACGCTGAGGTCAAGACCACCTACAAGGCCAAGAAGCCCGTGCAGCTGCCCGGCGCCTACAACGTCAACATCAAGTTGGACATCACCTCCCACAACGAGGACTACACCATCGTGGAACAGTACGAACGCGCCGAGGGCCGCCACTCCACCGGCGGCATGGACGAGCTGTACAAGTCTAGATCTGCCACCATGCTTGCATTTTGTTATTCGTTGCCCAATGCGGGcGATGTAATAAAGGGCAGAGTATACGAGAAGGATTATGCTCTATAcATTTATCTTTTTGACTATCCTCACTcTGAAGCTATCTTGGCAGAGAGTGTTAAGATGCATATGGATAGATATGTTGAATATAGGGATAAACTGGTAGGGAAAACTGTAAAAGTTAAAGTGATTAGAGTTGATTATACAAAAGGATATATAGATGTCAATTACAAAAGGATGTGTAGACATCAATAATAgGCGGCCGCCTAaacttgtttattgcagcttataatggttacaaataaagcaatagcatcacaaatttcacaaataaagcatttttttcactgcaAAAAAAAAAAAAAAAAAAAAAAAAAAAAAAAAAAAAAAAAAAAaaaaatattttagtgacaccatcagaaagaggtttaatatttttgtgagaccatcgaagagagaaagagaataaaaatatttttgtaaaactttttttatgagaccatcaaagagagaaagagaataaaaatatttttgtaaaactttttttatgagaccatcaaagagagaaagagaataaaaatatttttgtaaaactttttttatgagaccatcaaagagagaaagagaataaaaatatttttgtaaaactttttttatgagaccatcaaagagagaaagagaataaaaatatttttgtaaaactttttttatgagaccatcaaagagagaaagagaataaaaatatttttgtaaaactttttttatgagaccatcaaagagagaaagagaataaaaatatttttgtaaaactttttttatgagaccatcagaaagaggtttaatatttttgtgataccctgaaaggaaataggaataggaataggaatagtgtcataatcgtatcacactattgagacagaaaaagaagaagtaacgagaggtaactttttgtgaatgtagttaagaacatttttgttttgcaaaccggaatatagtgtccggtacacttttttaattcgtggtgtgcctgaatcgttcgattaaccctactcatccaatttcagatgaatagagttatcgattcagacacacgctttgagttttgttgaatcgatgagtgaagtatcatcggttgcaccttcagatgccgatccgtcgacatacttgaatccatccttgacctcaagttcagatgattccttgcacatgtctccgatacgaacgctaaactctagattcttgacacattttgtatcgacgatcgttgaaccgatgatatcttcgtaactcactttcttatgagagatgttagacccgagtactggatgggtcttgatgtcgctgtctttctcttcttcgctacatctgatgtcgatagacacctcacagtctttgatcatagccagagcttcttcacgagtgatcgcgggagagtccttaccttgtcctggggacacgctggacaatctagcattcactgtgtttccatcagcggattctgagatggatttaatctgaggacatttggtgaatccaaagttcattctcagacctccaccgatgatggagtaataagtggtaggaggatctacatcctcgactgatgtggaatcatcttctgattccacctcgggatctggatctgactcggactctgtaatttccgttacggattggcaaatcttatcattggtcggtgtttggtcttgctttgtgactttgataataacatcgattcccatatgatgtttgttttcttcttccgtacacgaggaggaggatgaggatgattgctgaagactggcaggcacatgcat

Isolate 5:

ttatagtataaagtaataaaaaatagttaatgtgatgactagcgccaccaacgccaacaacatttgataatttctacttactagacgtaccgtaaaaatataaattactataacaaataatagtatatcaataaacaacctaattaatggtcgaagtatagcaggacattgatgctctagaccgtgtataacaaaatctacaaatttttcatccgctatattttgtttcactatatcgtctagacgatcagcgataacttccatgttaatctattaaaatattatcaatatattttcagttttgcatatccgtggtagcaataaccatcggagaagttctaaagaatgtgtccatgtaagtagtccaatggacgttttccttattggctaaaataagtttgattttatcattggtggatgtgaacagcatacgcttggcatagtacataaacaacgctgccaatattataacaccgataacaatcatataaaactgaactcctgtaccagcaacttgtctaggtgctatttgagtagtggccttagtagtcaattgcatcaacgccttaatggcacaatttcctttgctagatcctgtattaataaattccaaatttgttggagatcctggggctccgtaacattcatctatgattacgttttgtatctttaatttgttatcgacgaccgcgctagaattacaagtttgttttacataattttcaaaatctctaacaacagtgtttacactcgtctgaatgtttaacgcagcagtaaacatagctggcacgtatgctttttgttccggtgttaatccactatatgtttctgtagcggctgataacacagcatccaactgagcatccgcgtccgcagagcacatatttttaacagtgaggttacatccatggttttgtcggatataaaaatttccgatttctatatcacattttgtttgagcactagcattcgcttcttgttctaatttagacgagatacgttcgctgagtgtattcaccgtcgtctgtatgcttgctgcggcacccatttaaatagctacaattagtatccatattaccaagagagataataaactgatcaaatgcaattttaggtcgaacgagtgtttaatattatgttgaactacttttgctctattgacgggaacagtagaaaatctatcactattgcttaatccacatgacaatcttaatgaggaagttttatccatctgtaagttgttcacgctagtattacatcgtacaatattgcaaagtcctaaattattataattacgtgttagcaagaaattaacattggcattcgaacactctggatcccaacattctcgaggttccgcatattttaatgactcttctaacttatctctagtgggataactacatctcatatatttctgtttaaagtccgcagactgttgtcttagaatataatcgatcatctctttgctatcttctgtattgtgtgcgcgtaaatgatgcaaaaatgattcacatattggtacactagcatctttactacataatgtttgcatcttattatacagatttattaacgattgttgaccctctacagttctattactcctattaaaggctgaaccgatccactgatggcatatgtttctatcgaacgtatccccctgacaccagtcgaataaatcaacatcgcattttccagtgtcgtgaacgtctggccaacacgattctaatactgcaccctcttcatacttatctgatatctttccatcctttttccaataatgagtacgattaaaagtgcgacagcaattgggtgcggttccattatacgattgtcttaacaatgccgaaagaccaccgggtcctgtgttgactaatctaaactctggatatcttttctttactaGctttttcgcaacgggtttgccgccagaacacagctgaagcttcgaggggctcgcatctctccttcacgcgcccgccgccctacctgaggccgccatccacgccggttgagtcgcgttctgccgcctcccgcctgtggtgcctcctgaactgcgtccgccgtctagCTCCTGGGCAACGGTACCGGATCCCTGCAGAAGCTTCTAAAAATTGAAATTTTATTTTTTTTTTTTGGAATATAAATGGTGAGCAAGGGCGAGGAGGATAACATGGCCATCATCAAGGAGTTCATGCGCTTCAAGGTGCACATGGAGGGCTCCGTGAACGGCCACGAGTTCGAGATCGAGGGCGAGGGCGAGGGCCGCCCCTACGAGGGCACCCAGACCGCCAAGCTGAAGGTGACCAAGGGTGGCCCCCTGCCCTTCGCCTGGGACATCCTGTCCCCTCAGTTCATGTACGGCTCCAAGGCCTACGTGAAGCACCCCGCCGACATCCCCGACTACTTGAAGCTGTCCTTCCCCGAGGGCTTCAAGTGGGAGCGCGTGATGAACTTCGAGGACGGCGGCGTGGTGACCGTGACCCAGGACTCCTCCCTGCAGGACGGCGAGTTCATCTACAAGGTGAAGCTGCGCGGCACCAACTTCCCCTCCGACGGCCCCGTAATGCAGAAGAAGACCATGGGCTGGGAGGCCTCCTCCGAGCGGATGTACCCCGAGGACGGCGCCCTGAAGGGCGAGATCAAGCAGAGGCTGAAGCTGAAGGACGGCGGCCACTACGACGCTGAGGTCAAGACCACCTACAAGGCCAAGAAGCCCGTGCAGCTGCCCGGCGCCTACAACGTCAACATCAAGTTGGACATCACCTCCCACAACGAGGACTACACCATCGTGGAACAGTACGAACGCGCCGAGGGCCGCCACTCCACCGGCGGCATGGACGAGCTGTACAAGTCTAGATCTGCCACCATGCTTGCATTTTGTTATTCGTTGCCCAATGCGGGcGATGTAATAAAGGGCAGAGTATACGAGAAGGATTATGCTCTATAcATTTATCTTTTTGACTATCCTCACTcTGAAGCTATCTTGGCAGAGAGTGTTAAGATGCATATGGATAGATATGTTGAATATAGGGATAAACTGGTAGGGAAAACTGTAAAAGTTAAAGTGATTAGAGTTGATTATACAAAAGGATATATAGATGTCAATTACAAAAGGATGTGTAGACATCAATAATAgGCGGCCGCCTAaacttgtttattgcagcttataatggttacaaataaagcaatagcatcacaaatttcacaaataaagcatttttttcactgcaAAAAAAAAAAAAAAAAAAAAAAAAAAAAAAAAAAAAAAAAAAAacttgtctaggtgctatttgagtagtggccttagtagtcaattgcatcaacgccttaatggcacaatttcctttgctagatcctgtattaataaattccaaatttgttggagatcctggggctccgtaacattcatctatgattacgttttgtatctttaatttgttatcgacgaccgcgctagaattacaagtttgttttacataattttcaaaatctctaacaacagtgtttacactcgtctgaatgtttaacgcagcagtaaacatagctggcacgtatgctttttgttccggtgttaatccactatatgtttctgtagcggctgataacacagcatccaactgagcatccgcgtccgcagagcacatatttttaacagtgaggttacatccatggttttgtcggatataaaaatttccgatttctatatcacattttgtttgagcactagcattcgcttcttgttctaatttagacgagatacgttcgctgagtgtattcaccgtcgtctgtatgcttgctgcggcacccatttaaatagctacaattagtatccatattaccaagagagataataaactgatcaaatgcaattttaggtcgaacgagtgtttaatattatgttgaactacttttgctctattgacgggaacagtagaaaatctatcactattgcttaatccacatgacaatcttaatgaggaagttttatccatctgtaagttgttcacgctagtattacatcgtacaatattgcaaagtcctaaattattataattacgtgttagcaagaaattaacattggcattcgaacactctggatcccaacattctcgaggttccgcatattttaatgactcttctaacttatctctagtgggataactacatctcatatatttctgtttaaagtccgcagactgttgtcttagaatataatcgatcatctctttgctatcttctgtattgtgtgcgcgtaaatgatgcaaaaatgattcacatattggtacactagcatctttactacataatgtttgcatcttattatacagatttattaacgattgttgaccctctacagttctattactcctattaaaggctgaaccgatccactgatggcatatgtttctatcgaacgtatccccctgacaccagtcgaataaatcaacatcgcattttccagtgtcgtgaacgtctggccaacacgattctaatactgcaccctcttcatacttatctgatatctttccatcctttttccaataatgagtacgattaaaagtgcgacagcaattgggtgcggttccattatacgattgtcttaacaatgccgaaagaccaccgggtcctgtgttgactaatctaaactctggatatcttttctttactttatcctctttatcttttgctagtggtcctatatgcacatattctaaaagcttagcgggagctatcacgtcatgcattttatccacgtttaataacatctcatcagtgggtactcccggaggcggatcccgtttagggagctcaacacttactccgccacccatatttatctcattgaaagtattaatctaaaaacgccataaagatgttgatcttaaaggattgaactctatccgaaaacaacattcctagaatgttatcgtcattatccattacgattctagtttcaaaaacattgactctctttttgaatcctcgtagtttgttgagagacgagatagctattttgaaagaaaacttttgtagttcttgagaacattcagtcatagaatattccctggaaaatgcatcagtattaccaggagtcttcataataatattgtcatctttaaacataatagccagatgctgatgctgactaatacacttgataaagcccaacaaccattctaaatgaatgacggttctaccacaacatttttcttcatacttgtgaaaattaaacacataagatttcttttgatctatacttagacaaatagtagtatctgtcctaataggcatcagttccttattacaatcgacacttactacatgataactagaaagttttactagattattttccagatcaggttctatatctatgatggcatcattgtgaaaactacacaaacacgattttaccttggacacaggaagattaaacacaatgttttcggctcctcggtagaacactgatgcactgagaggtataatggcccaaatgtttacagatccgcccaaggcggcaaaaatatacattaactcatccgtcgagtctacatttatagacacttcttcactgaactctgaaaaatatgccacaatttggcgcagtttatcgatttttatacggatgctcattttaaatttttgtaaattatttaaagttaaatggctgcagaacagcgtcgttctacaatttttgacatagtttcaaaatgtatagtgcaatctgtattaagagatatatctattaattctgaatacatagagtccaaagctaaacaattgtgctattgtccggcatcgaaaaaggaatcagtgattaatggtatctacaattgttgcgagtcaaatatagaaataatggacaaagagcagctattaaaaatattggacaatcttcgatgtcattcggctcatgtatgtaacgccacagatttctggagactatataattcgttaaaacggtttactcatactaccgcattctttaatacatgcaagcccactattctagccacg

Isolate 6:

atgccttctttgttctcctccctactaacgaccttagttttccatattttgatttattatcaaattaatttagtaactgtaaatataattatgaattgtttccaagaaaaacaattttcaagagaaaatttattaaaaatgccgtttagaatggttttaacgggaggatctggatctggaaaaactatctatttactatctctgttttctacactagttaaaaaatataaacatatattcttgtttacacccgtttataacccagattatgatggatacatttggccaaatcatattaatttcgttagtagtcaggaatctctagaatataatctgatacgaactaaaagtaacatagaaaaatgtattgctgtcgcacaaaatcataaaaaatcagcacactttttacttatttttgatgatgtaggcgataaactatcaaaatgcaatactctaatagaattcttaaactttggaaggcatttaaacacgtctattattctactatgccaaacttatagacacgtaccaatattaggacgggctaacattacgcatttttgtagttttaacatttccatctcagacgcggaaaatatgctacgatcgatgcctgtaaaggggaaacgaaaggatatattaaacatgttgaatatgatacagacagctagatccaataatcgattggctattattatcgaagactccgtattttgtgaaggtgaattacgtatatgtaccgataccgccgataaggacgtcatagaacaaaagttaaacatcgatattttagtaaatcaatattcgcacatgaaaaagaatctaaacgctatattagaaagtaaaaaaacctttttcgcaacgggtttgccgccagaacacagctgaagcttcgaggggctcgcatctctccttcacgcgcccgccgccctacctgaggccgccatccacgccggttgagtcgcgttctgccgcctcccgcctgtggtgcctcctgaactgcgtccgccgtctagCTCCTGGGCAACGGTACCGGATCCCTGCAGAAGCTTCTAAAAATTGAAATTTTATTTTTTTTTTTTGGAATATAAATGGTGAGCAAGGGCGAGGAGGATAACATGGCCATCATCAAGGAGTTCATGCGCTTCAAGGTGCACATGGAGGGCTCCGTGAACGGCCACGAGTTCGAGATCGAGGGCGAGGGCGAGGGCCGCCCCTACGAGGGCACCCAGACCGCCAAGCTGAAGGTGACCAAGGGTGGCCCCCTGCCCTTCGCCTGGGACATCCTGTCCCCTCAGTTCATGTACGGCTCCAAGGCCTACGTGAAGCACCCCGCCGACATCCCCGACTACTTGAAGCTGTCCTTCCCCGAGGGCTTCAAGTGGGAGCGCGTGATGAACTTCGAGGACGGCGGCGTGGTGACCGTGACCCAGGACTCCTCCCTGCAGGACGGCGAGTTCATCTACAAGGTGAAGCTGCGCGGCACCAACTTCCCCTCCGACGGCCCCGTAATGCAGAAGAAGACCATGGGCTGGGAGGCCTCCTCCGAGCGGATGTACCCCGAGGACGGCGCCCTGAAGGGCGAGATCAAGCAGAGGCTGAAGCTGAAGGACGGCGGCCACTACGACGCTGAGGTCAAGACCACCTACAAGGCCAAGAAGCCCGTGCAGCTGCCCGGCGCCTACAACGTCAACATCAAGTTGGACATCACCTCCCACAACGAGGACTACACCATCGTGGAACAGTACGAACGCGCCGAGGGCCGCCACTCCACCGGCGGCATGGACGAGCTGTACAAGTCTAGATCTGCCACCATGCTTGCATTTTGTTATTCGTTGCCCAATGCGGGcGATGTAATAAAGGGCAGAGTATACGAGAAGGATTATGCTCTATAcATTTATCTTTTTGACTATCCTCACTcTGAAGCTATCTTGGCAGAGAGTGTTAAGATGCATATGGATAGATATGTTGAATATAGGGATAAACTGGTAGGGAAAACTGTAAAAGTTAAAGTGATTAGAGTTGATTATACAAAAGGATATATAGATGTCAATTACAAAAGGATGTGTAGACATCAATAATAgGCGGCCGCCTAaacttgtttattgcagcttataatggttacaaataaagcaatagcatcacaaaAAAAAAAAAAAAAAAAAAAAAAAAAAAAAAAAAAAAAAAAAAAAAagaaagtaaaaaaacaaaattgtgcaatagcgatcaatcatcgtcgtcaaaaaatgtatcatcataattattaacttttgtaacaatagtcctatttagagaaagtctatcgatagacgatcccaatttgtaaattactccactacttgtcactccatgatatgataaatccatgtaaaatagcatcatctttagatcattaattgttaccttccccaatacaaccaaatcatcatgatatatacctcctccagacaagtatttaacaacggtagaatgctttggcttataaaatacaaatgacattcccttatgtttaatcttaatcttttctttagttattgaatcgttacaattataaaatgatgttttttccaaaaacctaagtgtatttaaaatagatgccat

Isolate 7:

atggagggatctaaacgcaaacacgatagtcggcgaccacaacaagaacaggagcagcctcgtccacgtacaccgccatcatatgaagaaattgcaaaatatggacactcatttaacgtgaaaagatttacgaatgaagaaatgtgtcttaagaatgattatccacgaGctttttcgcaacgggtttgccgccagaacacagctgaagcttcgaggggctcgcatctctccttcacgcgcccgccgccctacctgaggccgccatccacgccggttgagtcgcgttctgccgcctcccgcctgtggtgcctcctgaactgcgtccgccgtctagCTCCTGGGCAACGGTACCGGATCCCTGCAGAAGCTTCTAAAAATTGAAATTTTATTTTTTTTTTTTGGAATATAAATGGTGAGCAAGGGCGAGGAGGATAACATGGCCATCATCAAGGAGTTCATGCGCTTCAAGGTGCACATGGAGGGCTCCGTGAACGGCCACGAGTTCGAGATCGAGGGCGAGGGCGAGGGCCGCCCCTACGAGGGCACCCAGACCGCCAAGCTGAAGGTGACCAAGGGTGGCCCCCTGCCCTTCGCCTGGGACATCCTGTCCCCTCAGTTCATGTACGGCTCCAAGGCCTACGTGAAGCACCCCGCCGACATCCCCGACTACTTGAAGCTGTCCTTCCCCGAGGGCTTCAAGTGGGAGCGCGTGATGAACTTCGAGGACGGCGGCGTGGTGACCGTGACCCAGGACTCCTCCCTGCAGGACGGCGAGTTCATCTACAAGGTGAAGCTGCGCGGCACCAACTTCCCCTCCGACGGCCCCGTAATGCAGAAGAAGACCATGGGCTGGGAGGCCTCCTCCGAGCGGATGTACCCCGAGGACGGCGCCCTGAAGGGCGAGATCAAGCAGAGGCTGAAGCTGAAGGACGGCGGCCACTACGACGCTGAGGTCAAGACCACCTACAAGGCCAAGAAGCCCGTGCAGCTGCCCGGCGCCTACAACGTCAACATCAAGTTGGACATCACCTCCCACAACGAGGACTACACCATCGTGGAACAGTACGAACGCGCCGAGGGCCGCCACTCCACCGGCGGCATGGACGAGCTGTACAAGTCTAGATCTGCCACCATGCTTGCATTTTGTTATTCGTTGCCCAATGCGGGcGATGTAATAAAGGGCAGAGTATACGAGAAGGATTATGCTCTATAcATTTATCTTTTTGACTATCCTCACTcTGAAGCTATCTTGGCAGAGAGTGTTAAGATGCATATGGATAGATATGTTGAATATAGGGATAAACTGGTAGGGAAAACTGTAAAAGTTAAAGTGATTAGAGTTGATTATACAAAAGGATATATAGATGTCAATTACAAAAGGATGTGTAGACATCAATAATAgGCGGCCGCCTAaacttgtttattgcagcttataatggttacaaataaagcaatagcatcacaaaAAAAAAAAAAAAAAAAAAAAAAAAAAAAAAAAAAAAAAAAAAAAAaagaatgattatccacgaattatatcatataatcctccaccaaaatagagaatatatatatcatcatttcatgatgtatactactgacatagtttcaatgtgaacttttcactttcttgccggttatgaagaatattttttattttaatggtcattactaatcgtatattataattgaaaatgaattagtttaatatgacgctcgtcatgggattctgctgtggtagattctgtgacgctaagaataagaataagaataagaataagaataagaataagaataagaataagaaggaagatgtagaagagggaagagaaggatgttacaattataagaaccttaatgatctggatgaatccgaagcacgtgtagaatttggaccattatatatgataaatgaagaaaaatcagacataaatacattggatataaaaagaagatatagacacacgatagagtctgtatatttctaa

Isolate 8:

tgacaccagaaaacgacgaagagcagacatctgtgttctccgctactgtttacggagacaaaattcaaggaaagaataaacgcaaacgcgtgattggtctatgtattagaatatctatggttatttcactactatctatgattaccatgtccgcgtttctcatagtgcgcctaaatcaatgcatgtctgctaacgaggctgctattactgacgccgctgttgccgttgctgctgcatcatctactcatagaaaggttgcgtctactttttcgcaacgggtttgccgccagaacacagctgaagcttcgaggggctcgcatctctccttcacgcgcccgccgccctacctgaggccgccatccacgccggttgagtcgcgttctgccgcctcccgcctgtggtgcctcctgaactgcgtccgccgtctagCTCCTGGGCAACGGTACCGGATCCCTGCAGAAGCTTCTAAAAATTGAAATTTTATTTTTTTTTTTTGGAATATAAATGGTGAGCAAGGGCGAGGAGGATAAACATGGCCATCATCAAGGAGTTCATGCGCTTCAAGGTGCACATGGAGGGCTCCGTGAACGGCCACGAGTTCGAGATCGAGGGCGAGGGCGAGGGCCGCCCCTACGAGGGCACCCAGACCGCCAAGCTGAAGGTGACCAAGGGTGGCCCCCTGCCCTTCGCCTGGGACATCCTGTCCCCTCAGTTCATGTACGGCTCCAAGGCCTACGTGAAGCACCCCGCCGACATCCCCGACTACTTGAAGCTGTCCTTCCCCGAGGGCTTCAAGTGGGAGCGCGTGATGAACTTCGAGGACGGCGGCGTGGTGACCGTGACCCAGGACTCCTCCCTGCAGGACGGCGAGTTCATCTACAAGGTGAAGCTGCGCGGCACCAACTTCCCCTCCGACGGCCCCGTAATGCAGAAGAAGACCATGGGCTGGGAGGCCTCCTCCGAGCGGATGTACCCCGAGGACGGCGCCCTGAAGGGCGAGATCAAGCAGAGGCTGAAGCTGAAGGACGGCGGCCACTACGACGCTGAGGTCAAGACCACCTACAAGGCCAAGAAGCCCGTGCAGCTGCCCGGCGCCTACAACGTCAACATCAAGTTGGACATCACCTCCCACAACGAGGACTACACCATCGTGGAACAGTACGAACGCGCCGAGGGCCGCCACTCCACCGGCGGCATGGACGAGCTGTACAAGTCTAGATCTGCCACCATGCTTGCATTTTGTTATTCGTTGCCCAATGCGGGcGATGTAATAAAGGGCAGAGTATACGAGAAGGATTATGCTCTATAcATTTATCTTTTTGACTATCCTCACTcTGAAGCTATCTTGGCAGAGAGTGTTAAGATGCATATGGATAGATATGTTGAATATAGGGATAAACTGGTAGGGAAAACTGTAAAAGTTAAAGTGATTAGAGTTGATTATACAAAAGGATATATAGATGTCAATTACAAAAGGATGTGTAGACATCAATAATAgGCGGCCGCCTAaacttgtttattgcagcttataatggttacaaataaagcaatagcatcacaaaAAAAAAAAAAAAAAAAAAAAAAAAAAAAAAAAAAAAAAAAAAAagaaaggttgcgtctagcactacgcaatatgatcacaaagaaagctgtaatggtttatattaccagggttcttgttatatattacattcagactaccagttattctcggatgctaaagcaaattgcactgcggaatcatcaacactacccaataaatccgatgtcttgattacctggctcattgattatgttgaggatacatggggatctgatggtaatccaattacaaaaactacatccgattatcaagattctgatgtatcacaagaagttagaaagtatttttgtgttaaaacaatgaactaa

Isolate 9:

gtaaaattaaattaattataaaattatgtatatgatttactaactttagttagataagttagtaatacataaattttagtatattaatattatattttaaatattttatttagtgtctagaaaaaaatgtgtgaccaacgaccgtaggaaactctagagggtaagaaaaatcaatcgcGctttttcgcaacgggtttgccgccagaacacagctgaagcttcgaggggctcgcatctctccttcacgcgcccgccgccctacctgaggccgccatccacgccggttgagtcgcgttctgccgcctcccgcctgtggtgcctcctgaactgcgtccgccgtctagCTCCTGGGCAACGGTACCGGATCCCTGCAGAAGCTTCTAAAAATTGAAATTTTATTTTTTTTTTTTGGAATATAAATGGTGAGCAAGGGCGAGGAGGATAACATGGCCATCATCAAGGAGTTCATGCGCTTCAAGGTGCACATGGAGGGCTCCGTGAACGGCCACGAGTTCGAGATCGAGGGCGAGGGCGAGGGCCGCCCCTACGAGGGCACCCAGACCGCCAAGCTGAAGGTGACCAAGGGTGGCCCCCTGCCCTTCGCCTGGGACATCCTGTCCCCTCAGTTCATGTACGGCTCCAAGGCCTACGTGAAGCACCCCGCCGACATCCCCGACTACTTGAAGCTGTCCTTCCCCGAGGGCTTCAAGTGGGAGCGCGTGATGAACTTCGAGGACGGCGGCGTGGTGACCGTGACCCAGGACTCCTCCCTGCAGGACGGCGAGTTCATCTACAAGGTGAAGCTGCGCGGCACCAACTTCCCCTCCGACGGCCCCGTAATGCAGAAGAAGACCATGGGCTGGGAGGCCTCCTCCGAGCGGATGTACCCCGAGGACGGCGCCCTGAAGGGCGAGATCAAGCAGAGGCTGAAGCTGAAGGACGGCGGCCACTACGACGCTGAGGTCAAGACCACCTACAAGGCCAAGAAGCCCGTGCAGCTGCCCGGCGCCTACAACGTCAACATCAAGTTGGACATCACCTCCCACAACGAGGACTACACCATCGTGGAACAGTACGAACGCGCCGAGGGCCGCCACTCCACCGGCGGCATGGACGAGCTGTACAAGTCTAGATCTGCCACCATGCTTGCATTTTGTTATTCGTTGCCCAATGCGGGTGATGTAATAAAGGGCAGAGTATACGAGAAGGATTATGCTCTATATATTTATCTTTTTGACTATCCTCACTTTGAAGCTATCTTGGCAGAGAGTGTTAAGATGCATATGGATAGATATGTTGAATATAGGGATAAACTGGTAGGGAAAACTGTAAAAGTTAAAGTGATTAGAGTTGATTATACAAAAGGATATATcGATGTCAATTACAAAAGGATGTGTAGACATCAATAATAgGCGGCCGCCTAaacttgtttattgcagcttataatggttacaaataaagcaatagcatcacaaaAAAAAAAAAAAAAAAAAAAAAAAAAAAAAAAA……aaaaatcaatcgctttatagagaccatcagaaagaggtttaatatttttgtgagaccatcgaaggagaaagagataaaacttttttacgactccatcagaaagaggtttaatatttttgtgagaccatcgaaggagaaagagataaaacttttttacgactccatcagaaagaggtttaatatttttgtgagaccatcgaaggagaaagagataaaacttttttacgactccatcagaaagaggtttaatatttttgtgagaaaggagaaagagataaaacttttttacgactccatcagaaagaggtttaatatttttgtgagaccatcgaaggagaaagagataaaacttttttacgactccatcagaaagaggtttaatatttttgtgagaccatcgaaggagaaagagataaaacttttttacgactccatcagaaagaggtttaatatttttgtgagaccatcgaaggagaaagagataaaacttttttacgactccatcagaaagaggtttaatatttttgt

Isolate 10:

atggatatctttaaagaactaatcgtaaaacaccctgatgaaaatgttttgatttctccagtttctattttatctactttatctattctaaatcatggagcagctggttctacagctgaacaactatcaaaatatatagagaatatgaatgagaatacacccgatgacaataatgacatggacgtagatattccgtattgtgcgacactagctaccgcaaataaaatatacggtagcgatagtatcgagttccacgcctccttcctacaaaaaataaaagacgattttcaaactgtaaactttaataatgctaaccaaacaaaggaactaatcaacgaatgggttaagacaatgacaaatggtaaaattaattccttattgactagtccgctatccattaatactcgtatgacagttgttagcgccgtccattttaaagcaatgtggaaatatccattttctaaacatcttacatatacagacaagttttatatttctaagaatatagttaccagtgttgatatgatggtgggtaccgagaataacttgcaatatgtacatattaatgaattattcggaggattctctattatcgatattccatacgagggaaactctagtatggtgattatactaccggacgacatagaaggtatatataacatagaaaaaaatataacagatgaaaaatttaaaaaatggtgtggtatgttatctactaaaagtatagacttgtatatgccaaagtttaaagtggaaatgacagaaccgtataatctggtaccgattttagaaaatttaggacttactaatatattcggatattatgcagattttagcaagatgtgtaatgaaactatcactgtagaaaaatttctacatacgacgtttatagatgttaatgaggagtatacagaagcatcggccgttacaggagtatttacgattaacttttcgatggtatatcgtacgaaggtctacataaaccatccattcatgtacatgattaaagacaccacaggacgtatactttttatagggaaatactgctatccgcaataaatataaacaaatagacttttatcacgtttatctatgtctaaatattacaaatagtaatagtataaactaaagctgataatacttaaaaaaataataatatcatttacaattaatagtataaactaaaaattaaacaaatcgttattataagtaatatcaaaatgatgatatacggattaatagcgtgtcttatattcgtgacttcatccatcgctagtccacccattacggaagataaatcgttcaatagtgtagaggtattagtttccttgtttagagatgaccaaaaagactatacggtaacttctcagttcaataactacactatcgataccaaagactggactatccacacctgatggtttggatataccattgactaatataacttattggtcacggtttactataggtcgtgcattgttcaaatcagagtctgaggatattttccaaaagaaaatgagtattctaggtgtttctatagaatgtaagaagtcgtcgacattacttacttttttgaccgtgcgtaaaatgactcgagtatttaataaatttccagatatggcttattatcgaggagactgtttaaaagccgtttatgtaacaatgacttataaaaatactaaaactggagagactgattacacgtacctctctaatgggggttgcctgcatactatcgtaatggggtcgatggttgattattgattagtatattcctgctttttcgcaacgggtttgccgccagaacacagctgaagcttcgaggggctcgcatctctccttcacgcgcccgccgccctacctgaggccgccatccacgccggttgagtcgcgttctgccgcctcccgcctgtggtgcctcctgaactgcgtccgccgtctagCTCCTGGGCAACGGTACCGGATCCCTGCAGAAGCTTCTAAAAATTGAAATTTTATTTTTTTTTTTTGGAATATAAATGGTGAGCAAGGGCGAGGAGGATAACATGGCCATCATCAAGGAGTTCATGCGCTTCAAGGTGCACATGGAGGGCTCCGTGAACGGCCACGAGTTCGAGATCGAGGGCGAGGGCGAGGGCCGCCCCTACGAGGGCACCCAGACCGCCAAGCTGAAGGTGACCAAGGGTGGCCCCCTGCCCTTCGCCTGGGACATCCTGTCCCCTCAGTTCATGTACGGCTCCAAGGCCTACGTGAAGCACCCCGCCGACATCCCCGACTACTTGAAGCTGTCCTTCCCCGAGGGCTTCAAGTGGGAGCGCGTGATGAACTTCGAGGACGGCGGCGTGGTGACCGTGACCCAGGACTCCTCCCTGCAGGACGGCGAGTTCATCTACAAGGTGAAGCTGCGCGGCACCAACTTCCCCTCCGACGGCCCCGTAATGCAGAAGAAGACCATGGGCTGGGAGGCCTCCTCCGAGCGGATGTACCCCGAGGACGGCGCCCTGAAGGGCGAGATCAAGCAGAGGCTGAAGCTGAAGGACGGCGGCCACTACGACGCTGAGGTCAAGACCACCTACAAGGCCAAGAAGCCCGTGCAGCTGCCCGGCGCCTACAACGTCAACATCAAGTTGGACATCACCTCCCACAACGAGGACTACACCATCGTGGAACAGTACGAACGCGCCGAGGGCCGCCACTCCACCGGCGGCATGGACGAGCTGTACAAGTCTAGATCTGCCACCATGCTTGCATTTTGTTATTCGTTGCCCAATGCGGGcGATGTAATAAAGGGCAGAGTATACGAGAAGGATTATGCTCTATAcATTTATCTTTTTGACTATCCTCACTcTGAAGCTATCTTGGCAGAGAGTGTTAAGATGCATATGGATAGATATGTTGAATATAGGGATAAACTGGTAGGGAAAACTGTAAAAGTTAAAGTGATTAGAGTTGATTATACAAAAGGATATATAGATGTCAATTACAAAAGGATGTGTAGACATCAATAATAgGCGGCCGCCTAaacttgtttattgcagcttataatggttacaaataaagcaatagcatcacaaaATTCACAAATAAAGCATTTTTTCACTGCAAAAAAAAAAAAAAAAAAAAAAAAAAAaagagtaacagctgcccccattcttaataatcgtcagtatttaaactgttaaatgttggtatatcaacatctaccttatttcccgcagtataaggtttgttgcaggtatactgttcaggaatggttacatttatacttcttctatagtcctgtctttcgatgttcatcacatatgcaaagaacagaataaacaaaataatgtaagaaataatattaaatatctgtgaattcgtaaatacattgattgccataataattacagcagctacaatacacacaatagacattcccacagtgttgccattacctccacgatacatttgagttactaagcaataggtaataactaagctagtaagaggcaatagaaaagatgagataaatatcatcaatatagagattagaggagggctatatagagccaagacgaacaaaatcaaaccgagtaacgttctaacatcattatttttgaagattcccaaataatcattcattcctccataatcgttttgcatcatacctccatctttaggcataaacgattgctgctgttcctctgtaaataaatctttatcaagcactccagcacccgcagagaagtcgtcaagcatattgtaatatcttaaataactcat

Nanopore reads showing K3L-CNV:

Isolate 4: 4231d531-093c-4e3a-bf5f-ca4433a171d4

Isolate 5: e6dc6bc6-1905-411e-9712-db9dba137f55
