# Supplementary material for: Poxviruses capture host genes by LINE-1 retrotransposition
Source: eLife. 2022 Sep 7;11:e63332. doi: 10.7554/eLife.63332 (PMC9578705; doi:10.7554/eLife.63332)
Supplement: Transparent reporting form [file elife-63332-transrepform1.docx]

***eLife’s* transparent reporting form**

We encourage authors to provide detailed information *within their submission* to facilitate the interpretation and replication of experiments. Authors can upload supporting documentation to indicate the use of appropriate reporting guidelines for health-related research (see [EQUATOR Network](about:blank)), life science research (see the BioSharing Information Resource), or the [ARRIVE guidelines](about:blank) for reporting work involving animal research. Where applicable, authors should refer to any relevant reporting standards documents in this form.

If you have any questions, please consult our Journal Policies and/or contact us: [editorial@elifesciences.org](about:blank).

**Sample-size estimation**

- You should state whether an appropriate sample size was computed when the study was being designed
- You should state the statistical method of sample size computation and any required assumptions
- If no explicit power analysis was used, you should describe how you decided what sample (replicate) size (number) to use

Please outline where this information can be found within the submission (e.g., sections or figure legends), or explain why this information doesn’t apply to your submission:

This information doesn’t apply to our study, which involves standard phylogenetic analysis and devising a new experimental scheme to capture horizontal gene transfer events from host to virus genomes along with standard assays for virus replication.

**Replicates**

- You should report how often each experiment was performed
- You should include a definition of biological versus technical replication
- The data obtained should be provided and sufficient information should be provided to indicate the number of independent biological and/or technical replicates
- If you encountered any outliers, you should describe how these were handled
- Criteria for exclusion/inclusion of data should be clearly stated
- High-throughput sequence data should be uploaded before submission, with a private link for reviewers provided (these are available from both GEO and ArrayExpress)

Please outline where this information can be found within the submission (e.g., sections or figure legends), or explain why this information doesn’t apply to your submission:

The phylogenetic analysis shown in Figure 1B includes bootstrapping tests implemented in PhyML using maximum likelihood based phylogenetics. Details are provided in the figure legend and in the Methods section, “Phylogenetic analysis and sequence comparison of vGAAP.” Results from virus titer assays shown in Figures 2 & 4 include measurements of virus titers after 48 hours of cell infection. We performed three biological replicates by infecting 3 separate plates of each strain or isolate and measured virus titers at 48 hours. These data are shown as separate dots on each graph. At least two, but sometimes up to four technical replicates involving separate wells infected with the same biological replicate were averaged together to get these measurements. This information provided in the Methods section, “Measuring titers of virus strains and isolates.”

**Statistical reporting**

- Statistical analysis methods should be described and justified
- Raw data should be presented in figures whenever informative to do so (typically when N per group is less than 10)
- For each experiment, you should identify the statistical tests used, exact values of N, definitions of center, methods of multiple test correction, and dispersion and precision measures (e.g., mean, median, SD, SEM, confidence intervals; and, for the major substantive results, a measure of effect size (e.g., Pearson's r, Cohen's d)
- Report exact p-values wherever possible alongside the summary statistics and 95% confidence intervals. These should be reported for all key questions and not only when the p-value is less than 0.05.

Please outline where this information can be found within the submission (e.g., sections or figure legends), or explain why this information doesn’t apply to your submission:

Standard statistical analysis related to the phylogenetic analysis, including bootstrapping, was implemented in the PhyML package using default settings. We did no statistical comparisons between replication of virus isolates and strains other than displaying biological replicates and standard deviations.

(For large datasets, or papers with a very large number of statistical tests, you may upload a single table file with tests, Ns, etc., with reference to sections in the manuscript.)

**Group allocation**

- Indicate how samples were allocated into experimental groups (in the case of clinical studies, please specify allocation to treatment method); if randomization was used, please also state if restricted randomization was applied
- Indicate if masking was used during group allocation, data collection and/or data analysis

Please outline where this information can be found within the submission (e.g., sections or figure legends), or explain why this information doesn’t apply to your submission:

We did not group samples.

**Additional data files (“source data”)**

- We encourage you to upload relevant additional data files, such as numerical data that are represented as a graph in a figure, or as a summary table
- Where provided, these should be in the most useful format, and they can be uploaded as “Source data” files linked to a main figure or table
- Include model definition files including the full list of parameters used
- Include code used for data analysis (e.g., R, MatLab)
- Avoid stating that data files are “available upon request”

Please indicate the figures or tables for which source data files have been provided:

Our sequencing data is available at the NCBI SRA database: [http://www.ncbi.nlm.nih.gov/sra/PRJNA614958](about:blank)

This information is in the Methods under “Long read genome sequencing”.
